# Functional regulation of the structure-specific endonuclease FEN1 by the human cytomegalovirus protein IE1 suggests a role for the re-initiation of stalled viral replication forks

**DOI:** 10.1371/journal.ppat.1009460

**Published:** 2021-03-26

**Authors:** Eva-Maria Schilling, Myriam Scherer, Franziska Rothemund, Thomas Stamminger

**Affiliations:** Institute of Virology, Ulm University Medical Center, Ulm, Germany; University of Wisconsin-Madison, UNITED STATES

## Abstract

Flap endonuclease 1 (FEN1) is a member of the family of structure-specific endonucleases implicated in regulation of DNA damage response and DNA replication. So far, knowledge on the role of FEN1 during viral infections is limited. Previous publications indicated that poxviruses encode a conserved protein that acts in a manner similar to FEN1 to stimulate homologous recombination, double-strand break (DSB) repair and full-size genome formation. Only recently, cellular FEN1 has been identified as a key component for hepatitis B virus cccDNA formation. Here, we report on a novel functional interaction between Flap endonuclease 1 (FEN1) and the human cytomegalovirus (HCMV) immediate early protein 1 (IE1). Our results provide evidence that IE1 manipulates FEN1 in an unprecedented manner: we observed that direct IE1 binding does not only enhance FEN1 protein stability but also phosphorylation at serine 187. This correlates with nucleolar exclusion of FEN1 stimulating its DSB-generating gap endonuclease activity. Depletion of FEN1 and inhibition of its enzymatic activity during HCMV infection significantly reduced nascent viral DNA synthesis demonstrating a supportive role for efficient HCMV DNA replication. Furthermore, our results indicate that FEN1 is required for the formation of DSBs during HCMV infection suggesting that IE1 acts as viral activator of FEN1 in order to re-initiate stalled replication forks. In summary, we propose a novel mechanism of viral FEN1 activation to overcome replication fork barriers at difficult-to-replicate sites in viral genomes.

## Author summary

Human cytomegalovirus (HCMV) infection is usually asymptomatic in healthy individuals. However, newborns and immunocompromised patients are suffering from life-threatening diseases upon infection. After decades of research, vaccines are still not available and the use of antiviral therapeutics is limited. During the last years, drugs targeting the cellular DNA damage response (DDR) are increasingly applied in cancer therapy. Interestingly, HCMV utilizes the cellular DDR for its replication thereby implicating DDR components as promising targets for antiviral intervention. In this study, we found that the DDR enzyme flap endonuclease 1 (FEN1) is activated by the HCMV protein IE1 in a unique manner that depends on a direct protein-protein interaction. Moreover, we could demonstrate that FEN1, which is tremendously stabilized by IE1, supports efficient viral DNA replication. We propose a novel mechanism whereby a viral protein manipulates the cellular enzyme FEN1 to facilitate the multiplication of viral DNA genomes. Small molecule inhibitors of FEN1 might thus serve as a new antiviral therapy option.

## Introduction

The cellular DNA damage response (DDR) is a network of cellular pathways that sense, signal and repair DNA lesions arising from exogenous (e.g. UV radiation, ionizing radiation, genotoxic chemicals) as well as endogenous (e.g. reactive oxygen species, replication stress) sources. Depending on the type of DNA damage, different DDR signaling pathways are activated. While the kinase ATR (Ataxia telangiectasia and Rad3 related) mainly responds to DNA single strand breaks (SSB), the kinase ATM (Ataxia telangiectasia mutated) gets activated upon DNA double strand breaks (DSBs). DSBs, the most harmful type of DNA damage, can be repaired by homologous recombination (HR), non-homologous end joining (NHEJ) or single-strand annealing (SSA) depending on the cell cycle phase [1].

It is generally accepted that viral infections can trigger DDR, however, it is not completely understood whether this activation is a by-product of infection or actively induced by viral proteins. Moreover, it is not clear whether DDR factors facilitate or hinder viral replication. Some viruses have evolved strategies to circumvent or inhibit DDR, while others hijack cellular DNA repair proteins to facilitate the replication of their own genetic material (reviewed in [2–5]).

For human cytomegalovirus, a member of the subfamily of ß-herpesviruses, a robust response to DSBs centered on the activation of ATM and subsequent downstream signaling, meaning phosphorylation of ATM targets, has been observed in previous studies [3,6–8]. However, there are conflicting reports on whether the response to DSBs is required for productive viral replication. Gaspar and Shenk detected an activation of the major DSB-responding kinase ATM and its downstream targets. At later times post infection a cytoplasmic mislocalization of these factors was observed leading to the conclusion that HCMV escapes the consequences of DDR activation [6]. In contrast, more recent publications demonstrated that ATM protein expression and its kinase activity as well as activation of ATM downstream targets H2AX and p53 are necessary for an efficient HCMV replication [8,9]. Interestingly, the HCMV major immediate-early protein 1 (IE1) emerged as important player for the induction of the cellular DDR. This multifunctional key regulator, which is amongst the first proteins to be expressed upon infection, enables a successful HCMV infection by serving as antagonist of intrinsic and innate immune mechanisms, as promiscuous transactivator and as modulator of cell-cycle regulation and apoptosis [10]. Its role for stimulating the cellular DDR was first described by Castillo et al. showing that IE1 is sufficient for activation of ATM [11]. A later study could not only confirm this finding, but also demonstrated that the DSB marker γH2AX is likewise activated in an IE1-dependent manner [8]. Moreover, by utilizing a fluorescence-based double-stranded break repair assay, it has been demonstrated that IE1 can directly stimulate homology-directed repair [12].

A cellular factor that was recently identified to be involved in HR-mediated repair of stalled replication forks by actively inducing DSBs is Flap Endonuclease 1 (FEN1) [13]. FEN1 is described as key enzyme for maintaining genomic stability as it undertakes several functions during DNA replication and repair [14,15]. For this, FEN1 possesses three nuclease activities: Flap endonuclease-, 5’ exonuclease-, and the latest described gap endonuclease activity [16–18]. Flap endonuclease activity is required for RNA primer removal during lagging strand synthesis as well as for removal of flap structures formed during long-patch base excision repair. In contrast, a concerted action of 5’ exonuclease- and gap endonuclease activities produces DSBs, which are important for removal of hairpin structures, apoptotic DNA fragmentation as well as re-initiation of stalled replication forks. In order to control these multiple functions, FEN1 is tightly regulated by interplaying mechanisms: interaction with different protein partners, subcellular compartmentalization, and, most importantly, posttranslational modifications. The latter, in turn, is supposed to be important for the regulation of nuclease activities, protein partner selection and/or subcellular compartmentalization [16].

FEN1 is described to be acetylated, methylated, phosphorylated, SUMOylated, and ubiquitinated [19–21]. While acetylation reduces the overall enzymatic activity of FEN1 [19], methylation and phosphorylation both have stimulating effects on FEN1, even though on different enzymatic activities. Methylation of FEN1 at arginine 192 was shown to promote binding to the DNA sliding clamp PCNA, which in turn stimulates FEN1’s flap endonuclease activity, while FEN1 phosphorylation at serine 187 is simultaneously inhibited [22,23]. Meanwhile, an additional structural analysis could provide evidence that methylation at arginine 192 prevents FEN1 phosphorylation by preventing access of protein kinases [24]. Furthermore, by utilizing the FEN1 methylation mimic R192F, the authors could not only show that FEN1 phosphorylation and kinase binding were impaired but also its gap endonuclease activity [24]. In contrast, phosphorylation at serine 187 was shown to inhibit flap endonuclease activity by preventing PCNA binding [20]. All in all, Xu and colleagues proposed a model whereby methylation and phosphorylation antagonistically act on FEN1 by either promoting flap endonuclease or gap endonuclease activity, respectively [24].

Interestingly, phosphorylation of FEN1 stimulates its own SUMOylation [21,24]. While modification with SUMO3 was shown to stimulate FEN1 ubiquitination and subsequent proteasomal degradation in a cell cycle dependent manner, modification with SUMO1 prevents SUMO3-mediated degradation and promotes interaction of FEN1 with the Rad9–Rad1–Hus1 complex [21,25]. The Rad9–Rad1–Hus1 complex is a PCNA-like DNA sliding clamp that serves as platform to recruit DNA damage response and repair proteins to damaged DNA sites [26]. In accordance, SUMO1 modification of FEN1 emerged after UV irradiation and exposure to fork-stalling drugs hydroxyurea, camptothecin and mitomycin C [25]. Another work described that, likewise upon UV irradiation, FEN1 is excluded from the nucleolus, which was observed to be a phosphorylation-driven process [27]. Thus, the authors postulated that FEN1 is translocated out of nucleoli to participate in the re-initiation of stalled DNA replication forks [27].

In this report, we describe and characterize a novel interaction between HCMV IE1 and the structure-specific endonuclease FEN1. We observed that direct IE1 binding does not only enhance FEN1 protein stability but also induces phosphorylation at serine 187, which stimulates FEN1’s DSB-generating gap endonuclease activity, thereby proposing a kind of functional regulation. Consistently, a reduced/delayed induction of γH2AX, a sensitive marker for DSBs, was observed during HCMV infection in the absence of FEN1 thereby presumably reflecting a defect in re-initiating stalled viral replication forks. Consequently, viral DNA replication was markedly reduced in cells lacking FEN1.

## Results

### Yeast two-hybrid experiments identify the cellular DNA damage repair enzyme Flap endonuclease 1 as binding partner of HCMV IE1

**Fig 1:**
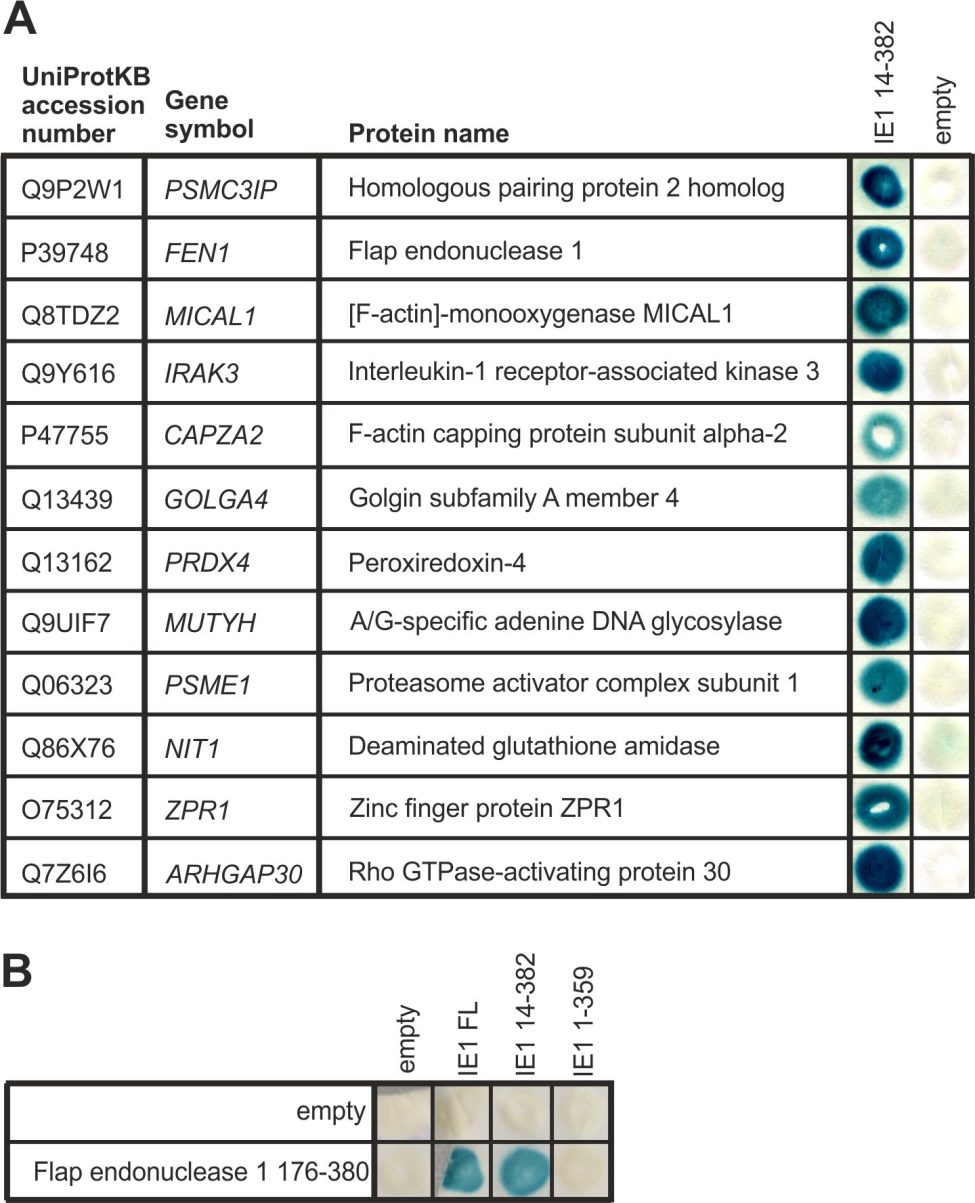
Identification of Flap endonuclease 1 as novel interactor of HCMV IE1. Yeast cells Y153 were transformed with two separate vectors, one of which encoded a GAL4 activation domain (AD) in fusion with the indicated cellular protein, the second plasmid encoded a GAL4 DNA-binding domain (DBD) in fusion with the respective IE1 versions. Thereafter, yeast colonies were selected for the presence of both plasmids on dropout medium lacking tryptophan and leucine and subsequently analyzed for the expression of β-galactosidase. (A) Interaction analysis between HCMV IE1 14–382, which serves as yeast two-hybrid screen bait, fused to the GAL4 DBD and the putative novel interactors as fusions with the GAL4 AD. (B) Interaction analysis between Flap endonuclease 1 in fusion to the GAL4 (AD) and HCMV IE1 as full length (FL) or truncated (14–382 and 1–359) versions as fusions with the GAL4 DBD.

To identify novel cellular binding partners of HCMV IE1, we performed yeast two-hybrid screening [28]. As bait, we used the globular core region of IE1 (IE1_CORE_), which comprises amino acids 14 to 382, in fusion with the DNA-binding domain of the transcription factor GAL4 in the pGBT9 vector backbone. A cDNA library derived from B lymphocytes fused to the activation domain of GAL4 in the vector pACT was selected as prey, as this library is highly complex and has already been used for the successful identification of interacting proteins [29–33]. By performing the screen as described in the “Material and Methods” section, we could identify 12 cellular proteins exhibiting an interaction with IE1 (Fig 1A). One of the identified proteins corresponded to a fragment (aa 176–380) of Flap endonuclease 1 (FEN1), a key enzyme for maintaining genomic stability by promoting not only DNA replication but also DNA damage repair [16,34]. As the activation of cellular DNA damage repair pathways is suggested to be indispensable for successful herpesviral DNA replication, we concentrated in this study on the characterization of FEN1 [2–5,35]. Therefore, we analyzed whether FEN1 176–380 likewise interacts with the full length version of IE1. For this, yeast two-hybrid analyses with FEN1 176–380 in fusion with the GAL4-AD and GAL4-BD fusions of either IE1_CORE_, IE1 full length (IE1 FL), or IE1_CORE_ with deletion of a C-terminal helix (IE1 1–359) were conducted (Fig 1B). FEN1 did not only bind to IE1_CORE_ but also to IE1 full length, while deletion of one single C-terminal helix of the core region abrogated the interaction, thereby identifying IE1_CORE_ as minimal requirement for FEN1 binding.

### Coimmunoprecipitation experiments confirm the interaction between IE1 and FEN1

**Fig 2:**
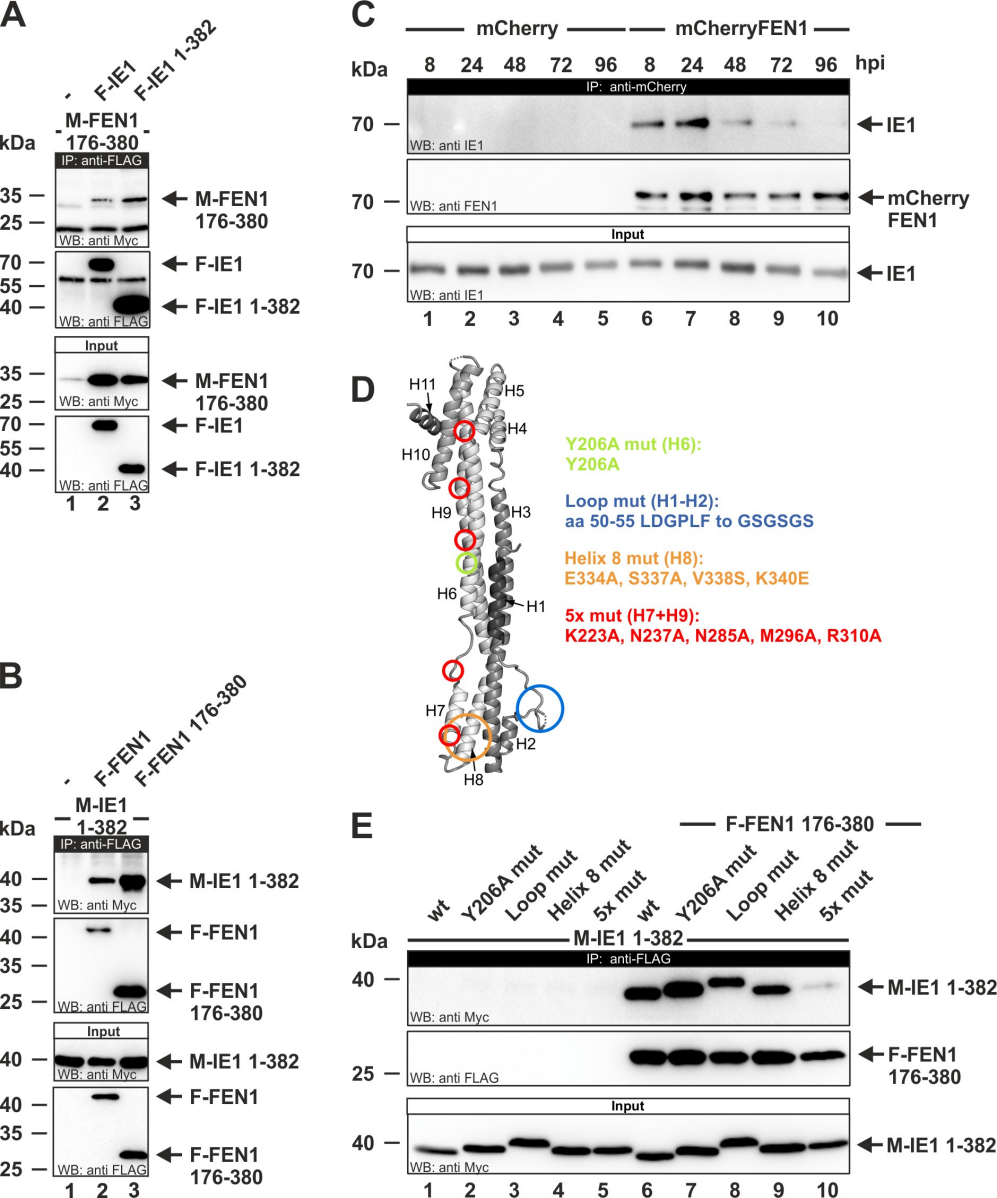
Characterization of the IE1-FEN1 interaction via coimmunoprecipitation (CoIP) experiments. (A, B, and E) HEK293T cells were cotransfected with the indicated Myc (M)- and FLAG (F)-tagged constructs. Upper two panels: Western blot detection of FEN1- and IE1 variants after immunoprecipitation (IP) with an anti-FLAG antibody. Lower panel/panels: detection of FEN1 and IE1 variants in cell lysates before precipitation (input). (A) IP of F-IE1 and F-IE1 1–382, CoIP of M-FEN1 176–380. (B) IP of F-FEN1 and F-FEN1 176–380, CoIP of M-IE1 1–382. (E) IP of F-FEN1 176–380, CoIP of different wildtype (wt) and mutant versions of M-IE1 1–382. (C) HFFs stably expressing mCherry or mCherryFEN1 were infected with AD169 at an MOI of 3 and lysed at the indicated times post infection. Upper two panels: Western blot detection of mCherryFEN1 and IE1 after immunoprecipitation (IP) with an anti-mCherry antibody. Lower panel: detection of IE1 in cells lysates before precipitation (input). (D) Schematic of the IE1_core_ structure. Regions that were mutated in order to generate Y206A, Loop mut, Helix 8 mut, and 5x mut are depicted in green, blue, orange, and red, respectively.

In order to confirm the data obtained in yeast, coimmunoprecipitation experiments were employed. For this, FEN1 176–380 was amplified via PCR and subcloned into mammalian expression vectors. The yielded Myc- and FLAG-tagged FEN1 constructs were utilized for coimmunoprecipitation experiments. HEK293T cells were cotransfected with Myc-FEN1 176–380 (M-FEN1 176–380) and FLAG-tagged versions of IE1 full length (F-IE1) and IE1_CORE_ (F-IE1 1–382). Cells transfected with M-FEN1 176–380 alone served as negative control. Two days later, the cells were harvested, lysed, and protein expression was analyzed by Western blotting using anti-FLAG or anti-Myc antibodies (Fig 2A, lower panels). Afterwards, FLAG-tagged proteins were immunoprecipitated using the anti-FLAG antibody. After separation of the protein complexes by SDS-PAGE, coprecipitated Myc-tagged proteins were detected by Western blotting (Fig 2A, first panel). FEN1 176–380 coprecipitated with both IE1_CORE_ as well as IE1 full length (Fig 2A, first panel, lanes 2 and 3). This precipitation was considered as specific since no signal was obtained when M-FEN1 176–380 was expressed alone (Fig 2A, lane 1). To confirm this, a reciprocal experiment was performed that additionally encloses the full length version of FEN1 (Fig 2B). For this, HEK293T cells were cotransfected with expression plasmids encoding FEN1 full length or FEN1 176–380 in fusion with a FLAG-tag (F-FEN1 and F-FEN1 176–380) and a Myc-tagged version of IE1_CORE_ (M-IE1 1–382). Cells transfected with IE1_CORE_ alone served as negative controls. The above described procedure was repeated and coprecipitated proteins were detected by Western blotting using anti-Myc antibody (Fig 2B, first panel). IE1_CORE_ was efficiently and specifically coprecipitated with FEN1 176–380 and also FEN1 full length. The negative control did not reveal any signal (Fig 2B, first panel, compare lanes 2 and 3 with 1). Having confirmed the newly identified interaction between IE1 and FEN1 in co-transfected cells, we asked whether there is also complex formation in HCMV-infected cells. For this, we generated primary human foreskin fibroblasts (HFFs) stably expressing mCherryFEN1 and, as control, mCherry alone thus enabling precipitation of FEN1 by utilizing an antibody directed against mCherry. The yielded cell populations were subsequently analyzed via immunofluorescence and Western Blot analyses (S1 Fig). In contrast to mCherry, which revealed a cytoplasmic staining pattern, mCherryFEN1 predominantly displayed a pan-nuclear staining pattern due to a nuclear localization signal at the C-terminus (S1A Fig, compare panels a to c with d to f) [36]. In order to assess the integrity of the overexpressed protein, Western blot analyses were performed by utilizing lysates of both cell lines (S1B Fig). The detection with an anti-FEN1 antibody revealed that both cell lines expressed endogenous FEN1 to equal amounts. An additional 70 kDa band emerged in cells stably expressing mCherryFEN1 representing the mCherry-tagged version of FEN1 (S1B Fig, lane 2). Subsequently, mCherry- as well as mCherryFEN1 cells were infected with the HCMV strain AD169 at a multiplicity of infection (MOI) of 3 and, at the indicated times post infection, harvested and lysed. After immunoprecipitating mCherry and mCherryFEN1, we analyzed the precipitates for the presence of IE1. As we could not detect any IE1 signal when precipitating the mCherry protein alone (Fig 2C, first panel, lanes 1 to 5), the coprecipitation of IE1 in infected mCherryFEN1 cells can be considered as specific. A complex consisting of IE1 and FEN1 could be detected starting at 8 hours post infection (hpi) (Fig 2C, first panel, lanes 6 to 10). Altogether, we could not only confirm the data obtained in yeast but also demonstrate a specific complex formation between FEN1 and IE1 in the viral context.

### Identification of a FEN1 binding deficient IE1 mutant

To further elucidate the mode of IE1-FEN1 interaction, we generated mutants of IE1_CORE_ in order to disrupt the binding between IE1 and FEN1. For this purpose, the IE1 structure was screened for regions of amino acids that are surface exposed and highly conserved among primate cytomegaloviruses. Mutations of exposed single amino acids or amino acid stretches, being distributed over the entire IE1 subdomain, were engineered and the resulting constructs were designated as Y206A mut, Loop mut, Helix 8 mut and 5x mut (Fig 2D). To study their binding to FEN1, we performed coimmunoprecipitation analyses after cotransfecting these Myc-tagged IE1_CORE_ variants with FLAG-FEN1 176–380. After immunoprecipitating FEN1 176–380 using the anti-FLAG antibody, we analyzed the precipitate for the presence of IE1_CORE_ variants. While Y206A mut, Loop mut and Helix 8 mut interacted with FEN1 176–380 comparably to the IE1_CORE_ wt (Fig 2E, first panel, compare lanes 7, 8 and 9 with 6), the 5x mut displayed a severely decreased interaction (Fig 2E, first panel, lane 10). Identification of this mutant allowed for a further detailed analysis of the IE1-FEN1 interplay.

### FEN1 is upregulated during HCMV infection in an IE1-dependent manner

**Fig 3:**
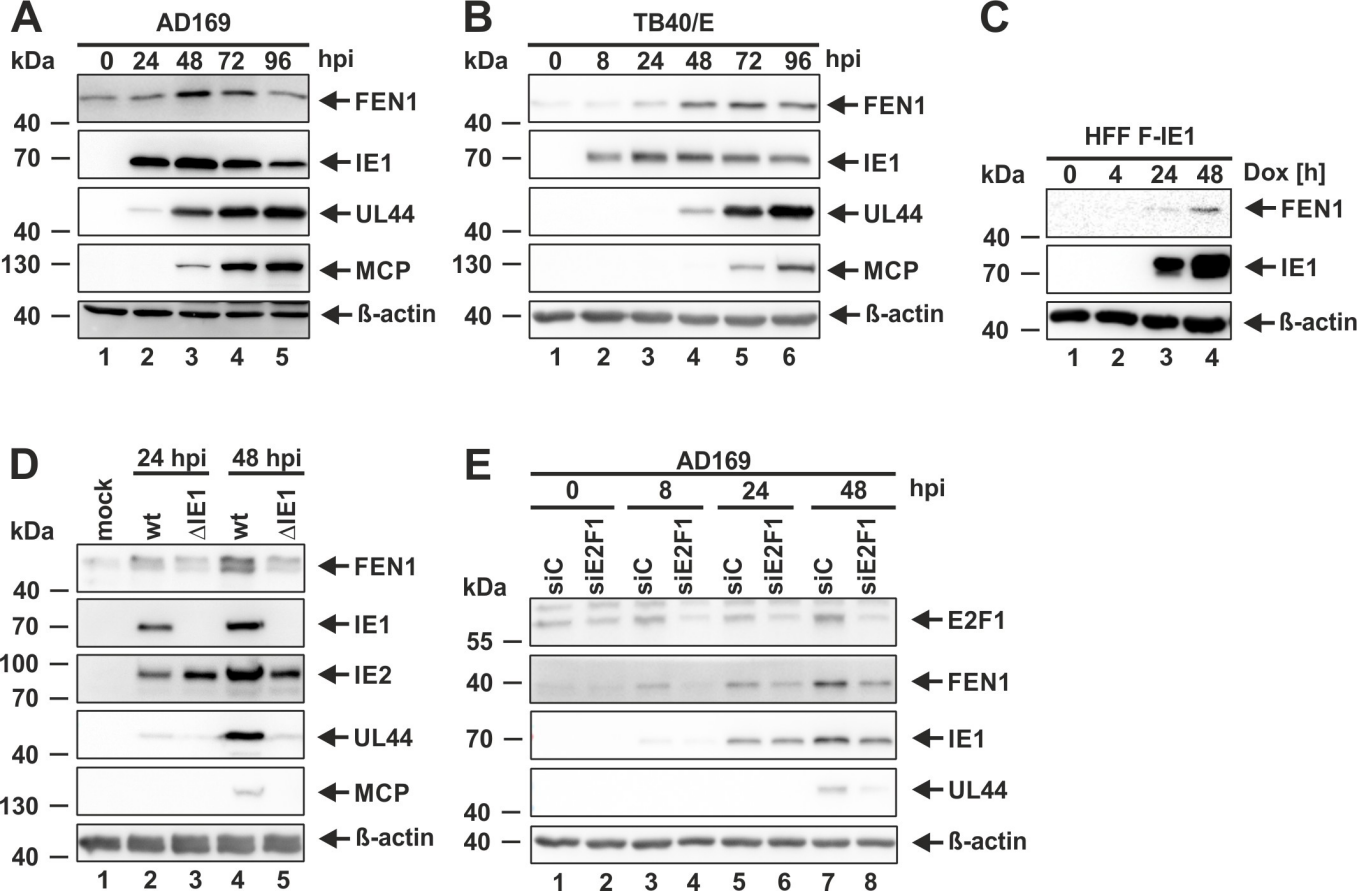
IE1-mediated upregulation of FEN1 during HCMV infection. (A, B, and D) HFF cells were either mock infected or infected with AD169 at an MOI of 3 (A), with TB40/E at an MOI of 1 (B), or with AD169 (wt) at an MOI of 7 and equivalent genome copy numbers of a recombinant strain lacking IE1 (ΔIE1) (D). At the indicated times post infection, cells were harvested and analyzed by Western blotting for FEN1, IE1, IE2 (for D), UL44, MCP, and β-actin. (C) HFF FLAG (F)-IE1 cells with doxycycline-inducible expression of FLAG (F)-IE1 were either not induced or induced with 0.5 μg/ml doxycycline for the indicated times. Cells were harvested analyzed by Western blotting for FEN1, IE1, and β-actin. (E) HFFs, which were treated for 24 h with 50 nM of a control siRNA (siC) or a siRNA targeting E2F1 (siE2F1), were infected with AD169 at an MOI of 3. At the indicated times post infection, cells were harvested and analyzed by Western blotting for E2F1, FEN1, IE1, UL44, and β-actin.

Next, we analyzed FEN1 protein levels throughout the viral replication cycle in order to identify a potential virus-mediated regulation of FEN1. For this, we infected HFFs with the HCMV strain AD169 at a MOI of 3 and analyzed endogenous FEN1 protein levels at different time points post infection by Western blotting (Fig 3A). The viral proteins IE1, UL44 and MCP served as marker for the immediate-early, early and late phase of infection, respectively. Starting at 24 hpi, we could observe an increase in FEN1 expression, that peaks at 48 hpi and declines with 72 hpi (Fig 3A, first panel). Interestingly, the expression of FEN1 seems to correlate with the expression level of IE1 that also decreases at 96 hpi (Fig 3A, second panel). In order to validate this finding with a less passaged HCMV strain, the same experiment was performed with the clinical isolate TB40/E at a MOI of 1. According to the AD169 infection, we could again detect an HCMV-mediated upregulation of FEN1 starting at 24 hpi (Fig 3B). To investigate whether IE1 is sufficient for the observed upregulation of FEN1 expression, we utilized HFFs with doxycycline-inducible expression of IE1 [37]. Remarkably, isolated IE1 expression induced the upregulation of FEN1 protein levels as it increases with IE1 accumulation starting at 24 hours post doxycycline addition (Fig 3C, first panel, lane 3). We next set out to investigate whether IE1 is required for the increase in FEN1 levels during HCMV infection (Fig 3D). To this end, we infected HFF cells with a high MOI of wild-type AD169 (wt) and equivalent genome copy numbers of a recombinant HCMV strain lacking IE1 (ΔIE1). At the indicated times post infection, cells were harvested and subjected to Western blot analyses. Intriguingly, we observed an attenuated accumulation of FEN1 protein levels at 24 and 48 hpi indicating that the presence of IE1 is necessary for an efficient upregulation of FEN1 during HCMV infection (Fig 3D, first panel, compare lanes 2 and 4 with 3 and 5). As IE1 is known to be an activator of the transcription factor E2F1 that has been shown to bind to the promoter region of FEN1, we consequently asked whether IE1 might upregulate FEN1 at the transcriptional level by utilizing E2F1. In order to answer this question, we transiently transfected HFFs with a small interfering RNA (siRNA) directed against E2F1 (siE2F1) or a non-targeting control siRNA (siC) and, 24 hours later, infected them with AD169 at a MOI of 3 (Fig 3E). Comparative time course analyses of siC or siE2F1 treated and infected cells revealed that there is a delayed but not abrogated accumulation of FEN1 suggesting that E2F1 contributes to FEN1 upregulation, however, additional mechanisms appear to be relevant (Fig 3D, panel 2, compare lanes 3, 5, and 7 with 4, 6, and 8).

### IE1 binding enhances FEN1 protein stability

**Fig 4:**
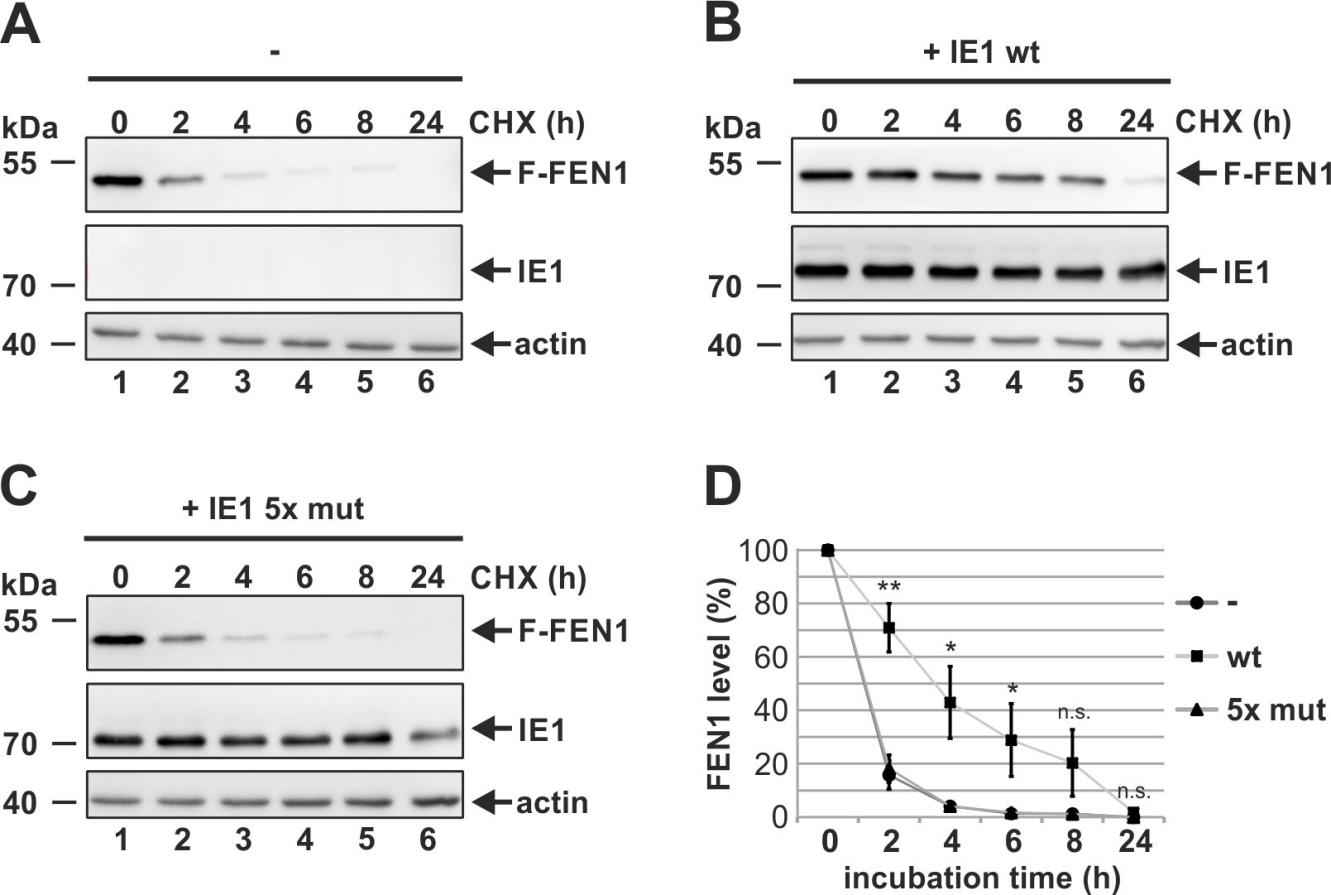
Stabilization of FEN1 by IE1 binding. (A, B, C, and D) 293T cells were transfected with FLAG (F)-FEN1 alone (A) or in combination with either IE1 wildtype (wt) (B) or IE1 5x mut (C). 18 hours post transfection, cells were treated with 10 μg/ml protein synthesis inhibitor cycloheximide (CHX) and harvested at the indicated times post addition. Western blot analyses were performed to detect FLAG (F)-FEN1, IE1, and β-actin. (D) Decline of FEN1 obtained by three independent experiments is represented by mean values ± SD. The p-values were calculated using two-tailed Student’s t-tests. n.s., not significant; **, p ≤ 0.01, *, p ≤ 0.05.

In order to further assess the mode of IE1-mediated FEN1 upregulation, we next analyzed whether IE1 influences FEN1 protein stability. For this, we transfected 293T cells with FLAG-FEN1 and examined its stability by applying the protein synthesis inhibitor cycloheximide (CHX). A subsequent Western blot analysis of FEN1 protein levels at different times post CHX addition identified FEN1 to be unstable as protein levels declined rapidly (Fig 4A, upper panel). At 2 hours post CHX addition, FEN1 was markedly reduced (Fig 4A, upper panel, compare lane 2 with 1), while it was almost completely degraded at 4 hours post CHX addition (Fig 4A, upper panel, lane 3). Surprisingly, FEN1 stability was considerably increased in the presence of IE1 wt (Fig 4B, upper panel). Protein levels remained stable until 4 hours post CHX addition and declined slowly (Fig 4B, upper panel, compare lanes 2, 3, 4 and 5 with lane 1). In contrast, the FEN1 binding-deficient mutant IE1 5x mut was incapable of increasing FEN1 stability (Fig 4C, upper panel). Densitometric analyses of three independent experiments revealed that the decline of FEN1 in the presence of IE1 5x mut resembled that without IE1, while the presence of IE1 wt tremendously enhanced FEN1 stability (Fig 4D). Taken together, HCMV induces an upregulation of FEN1 protein levels which appears to be mediated by two different mechanisms: transcriptional activation of FEN1 expression via transcription factor E2F1 and a strong increase of FEN1 protein stability which requires a direct binding to IE1.

### FEN1 acts in a proviral way by promoting HCMV genome replication

**Fig 5:**
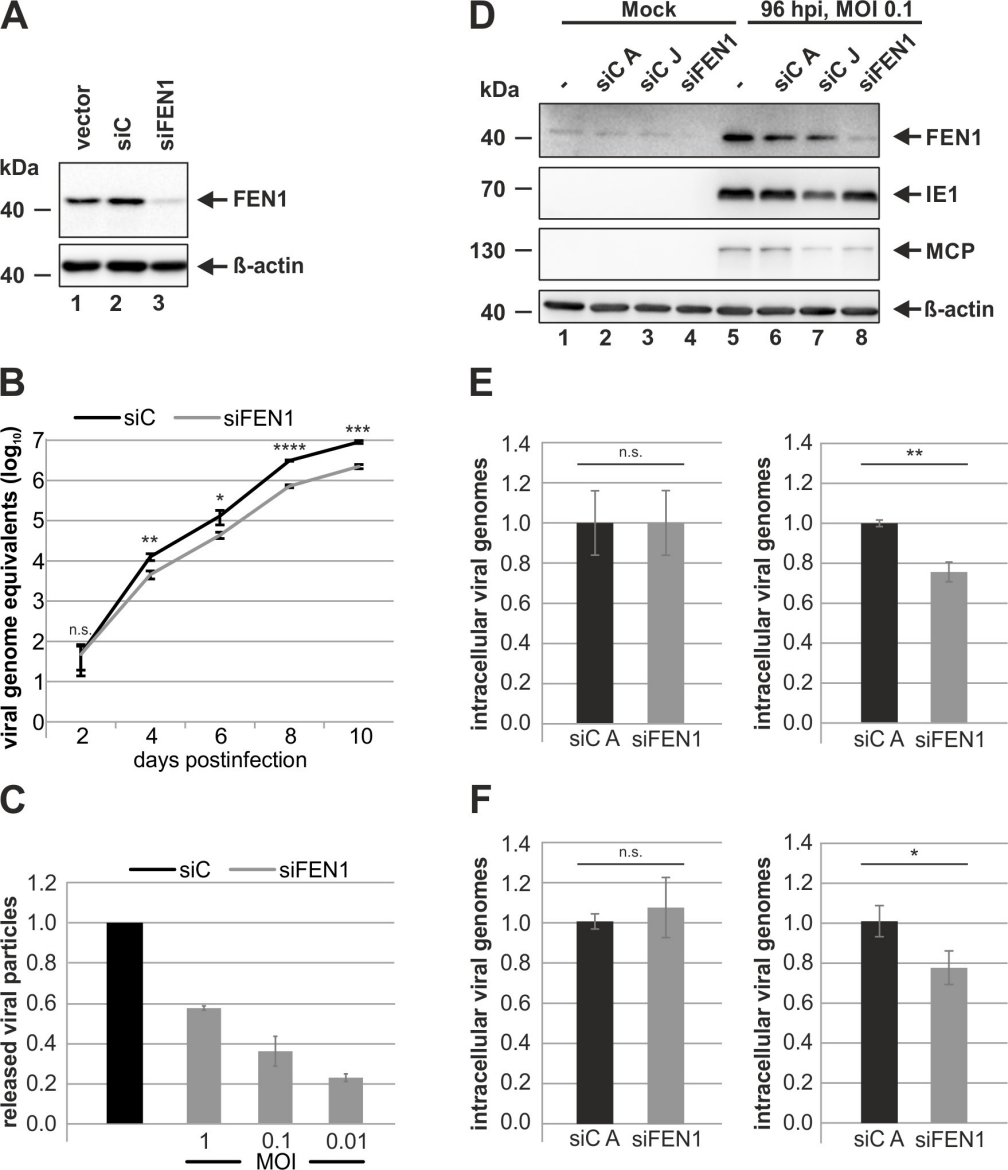
Promotion of viral DNA replication by FEN1. (A) Detection of FEN1 protein levels in vector-, siC-, and siFEN1-transduced HFFs by Western blotting. β-actin levels served as loading control. (B and C) siC- and siFEN1-transduced HFFs were infected at an MOI of 0.01 (B) or at indicated MOIs (C) and multistep growth curve analysis (B) or virus release assay (C) was performed. Cell supernatants were harvested at the indicated times after infection and analyzed for viral genome equivalents by HCMV IE1-specific quantitative real-time PCR. For C, siC-transduced HFFs were set to 1. Values are derived from biological triplicates and represent mean values ± SD. (D) HFF cells were either transfected with two control siRNAs (siC A and siC J) or siRNA directed against FEN1 or left untreated. 24 h later, cells were either directly harvested (Mock) or infected with AD169 at an MOI of 0.1 and harvested 96 hpi. Cells were analyzed by Western blotting for FEN1, IE1, MCP, and β-actin. (E and F) siC A- and siFEN1-transfected HFFs were infected with AD169 (E) or TB40/E (F) at an MOI of 0.1, and total DNA was extracted at 8 hpi (left) and 96 hpi (right) using the DNeasy blood and tissue kit (Qiagen). Viral genomes were quantified by TaqMan real-time PCR specific for IE1, and genome copy numbers were calculated. siC A-transfected HFFs were set to 1. Values are derived from biological triplicates and represent mean values ± SD. For panels B, E, and F, the p-values were calculated using two-tailed Student’s t-tests. n.s., not significant; ****, p ≤ 0.0001; ***, p ≤ 0.001; **, p ≤ 0.01; *, p ≤ 0.05.

As the IE1-mediated increase in FEN1 protein levels indicated that FEN1 might be beneficial for HCMV, we set out to examine the exact role of FEN1 for viral replication. For this, we generated cells with a partial depletion of FEN1, which was accomplished by retrovirally transducing HFFs with a vector encoding an shRNA directed against FEN1 transcripts. The yielded cell population stably expressing the shRNA was termed HFF siFEN1. Control cell populations, which were retrovirally transduced with either a vector encoding non-targeting siRNA or an empty vector, were equally generated and termed HFF siC or HFF vector, respectively. As seen in Fig 5A, FEN1 protein levels were decreased in cells expressing FEN1 shRNA (Fig 5A, first panel, compare lane 3 with lanes 1 and 2). Next, we analyzed the growth kinetics of AD169 in HFF siFEN1 and HFF siC by performing comparative multistep growth curve analysis at a MOI of 0.01 (Fig 5B). For this purpose, HFF cells were infected and virus-containing cell supernatants were harvested at the indicated times post infection. Subsequently, the supernatants were subjected to quantitative real-time PCR to determine HCMV genome equivalents. HCMV revealed a delayed growth in siFEN1- in comparison to siC cells thereby indicating that the loss of FEN1 creates an unfavorable environment for HCMV replication. In order to analyze whether the effect of FEN1 knockdown on HCMV growth is MOI-dependent, we quantified viral release in the absence of FEN1 at MOIs of 1, 0.1, and 0.01 (Fig 5C). Strikingly, we could observe a clear MOI-dependence thus indicating that FEN1 is required for an efficient HCMV growth especially at low MOI conditions (Fig 5C, compare bars 2 to 4 with 1). It is generally proven that FEN1 is implicated in several processes at the replication fork to promote cellular DNA replication [16,34]. Consequently, we continued to analyze the impact of FEN1 on viral DNA replication. As we observed only a partial loss of FEN1 in HFFs stably expressing shRNA against FEN1 transcripts, we pursued another strategy by transiently transfecting siRNA directed against FEN1. Efficient FEN1 knockdown could be verified by Western blotting in uninfected cells as well as in AD169-infected HFFs (Fig 5D, first panel, lane 4 and 8). As control, we utilized two different siRNAs namely siC A and siC J. By comparing these control siRNAs, we decided to continue further experiments with siC A as siC J already caused diminished viral gene expression (Fig 5D, lane 7). Next, we infected HFFs, which were transfected with either control siRNA (siC A) or siRNA against FEN1 (siFEN1), at a MOI of 0.1 and isolated intracellular viral genomes at 8 hpi and 96 hpi in order to determine HCMV genome equivalents by quantitative real-time PCR (Fig 5E). Despite equal input genomes at 8 hpi, we could detect a significant reduction in intracellular HCMV genomes at 96 hpi in cells treated with siFEN1 (Fig 5E, compare left two bars with right two bars). The same result was obtained when repeating the experiment under identical conditions with the clinical isolate TB40/E thereby excluding a virus strain specific effect (Fig 5F). Collectively, these findings strongly suggest that FEN1 facilitates HCMV growth by promoting HCMV DNA replication.

### Enzymatic activity of FEN1 is required for efficient HCMV genome replication

**Fig 6:**
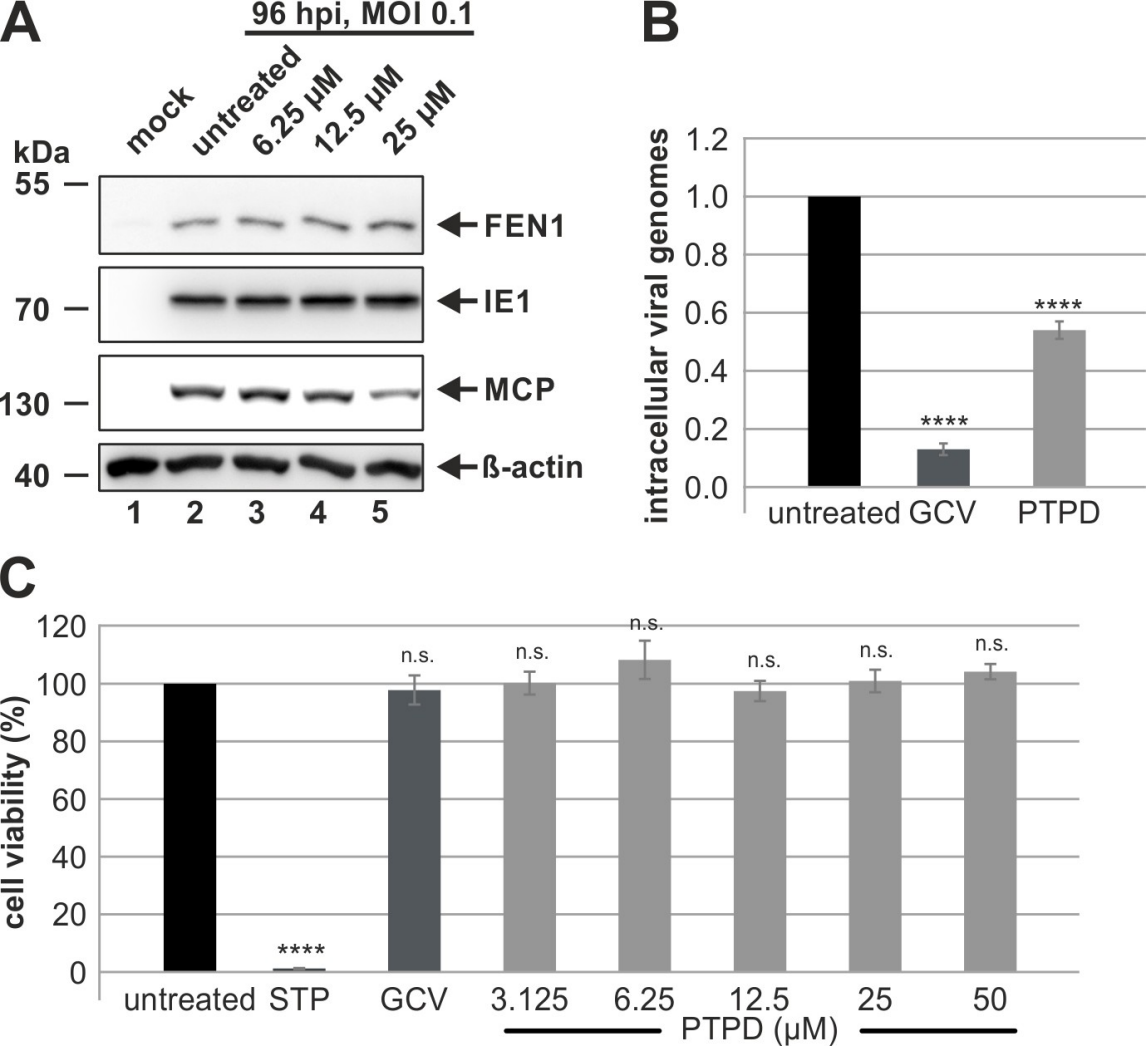
Requirement of FEN1’s enzymatic activity for viral DNA replication. (A) HFFs were infected with AD169 at an MOI of 0.1 or left uninfected (mock) and, at 2 hpi, treated either with DMSO or with different concentrations PTPD. At 96 hpi, cells were harvested and analyzed by Western blotting for FEN1, IE1, MCP, and β-actin. (B) HFFs were infected with AD169 at an MOI of 0.1 and, at 2 hpi, treated with the FEN1 inhibitor PTPD (25 μM), ganciclovir (GCV) (20 μM) or left untreated. At 96 hpi, total DNA was extracted, viral genomes were quantified by TaqMan real-time PCR specific for IE1, and genome copy numbers were calculated. Untreated cells were set to 1. Values are derived from biological triplicates and represent mean values ± SD. (C) Cell viability measured after a 96 h treatment of HFFs with the positive control Staurosporin (STP) (5 μM), ganciclovir (GCV) (20 μM) and PTPD (concentrations as indicated). Untreated cells were set to 100%. Values are derived from biological triplicates and represent mean values ± SD. For panels B and C, the p-values were calculated using two-tailed Student’s t-tests. n.s., not significant; ****, p ≤ 0.0001.

To fulfill its function at the cellular genome, FEN1 needs to be enzymatically active [16]. Therefore, we continued to elucidate whether enzymatic activity of FEN1 is likewise required for viral DNA replication by applying the FEN1 inhibitor 3-hydroxy-5-methyl-1-phenylthieno [2, 3-d] pyrimidin-e-2, 4(1H, 3H) dione (PTPD) [38,39]. In order to confirm that the substance does not alter FEN1 expression but only its enzymatic activity, FEN1 protein levels were analyzed during HCMV infection in the presence of PTPD (Fig 6A). This excluded variations in FEN1 expression by applying PTPD (Fig 6A, first panel). In addition, we could already observe inhibiting effects on viral DNA replication. While IE1 (Fig 6A, second panel), whose expression does not require DNA *de novo* synthesis, remained unchanged in the presence of PTPD, expression of the late protein MCP (Fig 6A, third panel) was decreased in a concentration-dependent manner. Next, we repeated the experiment as depicted in Fig 5E but utilized the FEN1 inhibitor instead of siRNA against FEN1. To avoid any impact on HCMV entry, we added PTPD as well as the positive control ganciclovir (GCV) at 2 hpi and isolated intracellular viral genomes at 96 hpi to assess HCMV genome equivalents by quantitative real-time PCR (Fig 6B). Strikingly, an approximately 2-fold reduction in intracellular HCMV genomes could be detected in cells treated with PTPD, which is in accordance with the siRNA experiments (see Fig 5E and 5F). Finally, we excluded that the observed effect was due to any toxicity by determining cell viability in the presence of PTPD (Fig 6C). Altogether, by utilizing the FEN1 inhibitor PTPD, we could demonstrate that FEN1 requires its enzymatic activity to promote HCMV DNA replication.

### IE1 evokes nucleolar exclusion of FEN1

**Fig 7:**
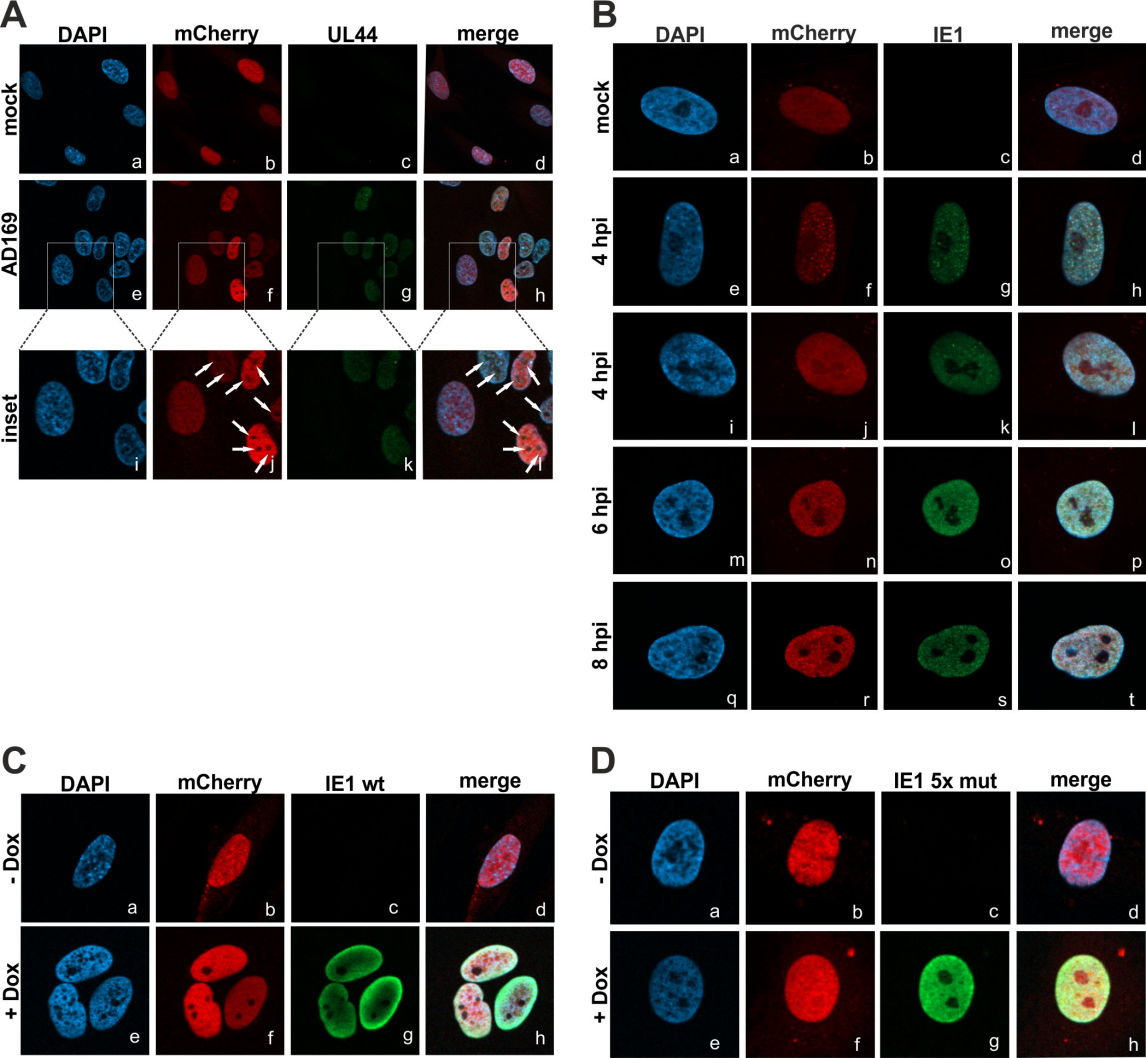
IE1-mediated nucleolar exclusion of FEN1 during HCMV infection. (A) mCherryFEN1 cells were infected with AD169 at an MOI of 1 and, at 24 hpi, analyzed by detecting the red fluorescent protein mCherry as well as UL44 as marker for infected cells by utilizing an antibody directed against UL44. Nucleoli are marked by arrowheads in the inset images. (B) mCherryFEN1 cells were infected with AD169 at an MOI of 1 and, at indicated time points, analyzed by detecting the red fluorescent protein mCherry as well as IE1 by utilizing an antibody directed against IE1. (C and D) Doxycycline-inducible HFF IE1 wildtype (wt) (C) and HFF IE1 5x mut (D) were transduced with mCherryFEN1. Cells treated (+ Dox) or untreated (- Dox) with doxycycline (0.5 μg/ml) were analyzed by detecting the red fluorescent protein mCherry as well as IE1 by utilizing an antibody directed against IE1.

FEN1 is described to regulate its multiple functions via different mechanisms. Beside its regulation via posttranslational modifications and protein-protein interactions, FEN1 is additionally controlled by cellular compartmentalization [16]. In order to analyze the subcellular localization of FEN1 during HCMV infection, we again utilized mCherry- and mCherryFEN1 expressing HFFs (see S1 Fig). In order to analyze whether HCMV alters the localization of FEN1, mCherryFEN1 cells were infected with AD169 and examined via indirect immunofluorescence utilizing UL44 as marker for infection (Fig 7A). Interestingly, upon AD169 infection, all cells infected with HCMV displayed a changed FEN1 staining pattern (Fig 7A, panels e to h): FEN1 was observed to be excluded from a subnuclear structure (marked by arrowheads), which presumably corresponds to the nucleolus (Fig 7A, panels i to l). To further investigate the observed exclusion of FEN1, we again infected HFFs mCherryFEN1 with AD169 and analyzed earlier time points reflecting the immediate-early phase of infection (Fig 7B). At 4 hpi, there were two different FEN1 staining patterns visible: one subgroup of cells (Fig 7B, panels e to h) displayed a dot-like co-localization between FEN1 and IE1, the other subgroup (Fig 7B, panels i to l) revealed a more dispersed FEN1 staining pattern thereby again resembling IE1 distribution. Collectively, both subgroups exhibited nucleolar exclusion of FEN1, which was more prominent at 6 hpi (Fig 7B, panels m to p) and 8 hpi (Fig 7B, panels q to t). Next, we analyzed whether IE1 is sufficient to induce this translocation event, as the exclusion was shown to already start during the immediate-early phase of infection (see Fig 7B). For this, we utilized HFFs with doxycycline-inducible expression of IE1 wildtype (wt) and additionally transduced them with lentiviruses expressing mCherryFEN1 (Fig 7C). Strikingly, upon induction of IE1 wt expression, FEN1 displayed nucleolar exclusion (Fig 7C, panels e to h). In contrast, when repeating this experiment utilizing HFFs with doxycycline-inducible expression of the FEN1 binding-deficient mutant IE1 5x mut (see Fig 2D and 2E), nucleolar exclusion of FEN1 was not detected (Fig 7D, panels e to h). Taken together, our experiments demonstrated that IE1 is sufficient to induce a nucleolar exclusion of FEN1, which requires a direct protein-protein interaction.

### IE1 induces accumulation of FEN1 phosphorylated at serine 187

**Fig 8:**
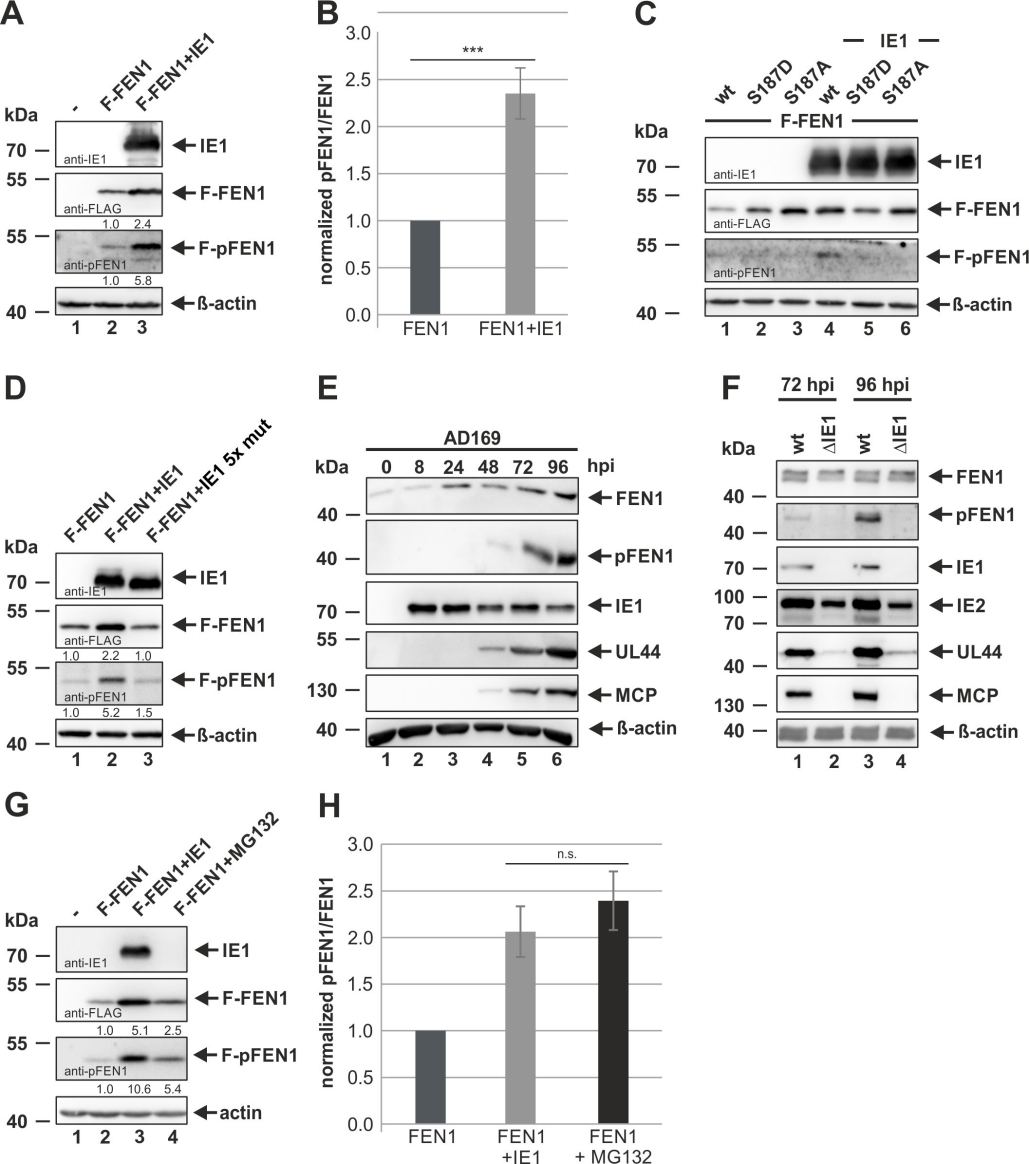
Accumulation of phosphorylated FEN1 species upon IE1 expression. (A, B, C, D, G and H) 293T cells were transfected with the indicated constructs and, at 42 h post transfection, treated with MG132 (5 μM) when indicated. 48 h post transfection, cells were harvested and analyzed by Western blotting for IE1, FLAG, pFEN1 (Ser187), and β-actin. (A, B, D, G and H) Protein levels of FEN1 and pFEN1 were quantified via densitometric analyses utilizing the Image Lab Software. Protein levels without IE1/MG132 were set to 1. (B and H) Ratios between pFEN1 and FEN1 obtained by three independent experiments are represented by mean values ± SD. The p-values were calculated using two-tailed Student’s t-test. n.s., not significant; ***, p ≤ 0.001. (E and F) HFF cells were either mock infected or infected with AD169 at an MOI of 3 (E) or with AD169 (wt) at an MOI of 7 and equivalent genome copy numbers of a recombinant strain lacking IE1 (ΔIE1) (F). At the indicated times post infection, cells were harvested and analyzed by Western blotting for FEN1, pFEN1, IE1, IE2 (for F), UL44, MCP, and β-actin.

As nucleolar exclusion of FEN1 is described to depend on its phosphorylation at serine 187 [27], we pursued to analyze the phosphorylation state of FEN1 in the presence of IE1. For this, we utilized a phospho-specific antibody (anti-pFEN1), which enables to exclusively detect FEN1 species phosphorylated at serine 187. 293T cells were transfected with a plasmid expressing FLAG-FEN1 with either an empty control vector or a plasmid expressing IE1. Two days later, whole cell lysates were analyzed by Western blotting using anti-IE1, anti-FLAG and anti-pFEN1 antibodies (Fig 8A). Intriguingly, in the presence of IE1, we could detect a strong signal with the anti-pFEN1 antibody thereby indicating that IE1 mediates phosphorylation of FEN1 at serine 187 (Fig 8A, third panel, lane 3). As pan-FEN1 was also increased 2.4-fold in the presence of IE1 (Fig 8A, second panel, lane 3), we conducted three independent experiments to exclude that the accumulation of phosphorylated FEN1 species is merely a by-product of this overall increase. Subsequent densitometric analyses revealed that the increase of phosphorylated FEN1 species was approximately 2.4 higher than the increase of panFEN1 thereby providing evidence that there is a specific accumulation of FEN1 phosphorylation in the presence of IE1 (Fig 8B). To confirm antibody specificity, we repeated the experiment and included FLAG-tagged FEN1 constructs expressing either a phosphomimic (S187D) or a phosphomutant (S187A), both deficient in phosphorylation at serine 187 (Fig 8C). By comparing these constructs with FEN1 wt, it could be clearly demonstrated that the antibody exclusively detects FEN1 phosphorylated at serine 187 as there was no band detectable after expressing both phosphorylation deficient mutants (Fig 8C, compare lanes 5 and 6 with lane 4). In order to analyze the dependence of FEN1 phosphorylation on IE1 binding, we utilized the FEN1 binding-deficient mutant IE1 5x mut for an additional transfection experiment (Fig 8D). For this, FLAG-FEN1 was transfected alone or in combination with either the IE1 wt or the IE1 5x mut. In the presence of IE1 wt, we could again detect a normalized pFEN1/FEN1 ratio of 2.4, while the IE1 5x mut only slightly affected FEN1 phosphorylation as demonstrated by a ratio of 1.5 (Fig 8D). To make sure that the observed phosphorylation of FEN1 also occurs in the viral context, we next investigated the phosphorylation of endogenous FEN1 during the time course of HCMV infection (Fig 8E). Therefore, we infected HFFs with AD169, harvested the cells at indicated time points and followed FEN1 phosphorylation by again utilizing the anti-pFEN1 antibody. As expected, starting at 48 hpi, phosphorylation of FEN1 at serine 187 was detected (Fig 8E, second panel, lane 4 to 6). As we could already demonstrate that IE1 is sufficient for the accumulation of phosphorylated FEN1 species (see Fig 8A and 8B), we asked whether IE1 is also necessary for the increase of phosphorylated FEN1 during HCMV infection (Fig 8E). To answer this issue, we infected HFF cells with a high MOI of wild-type AD169 (wt) and equivalent genome copy numbers of a recombinant HCMV strain lacking IE1 (ΔIE1) and analyzed pFEN1 at late times of infection. Western Blot analyses clearly revealed that IE1 is required for the accumulation of phosphorylated FEN1, as we could not detect pFEN1 during infection with the IE1-deleted virus (Fig 8F, first panel, compare lanes 1 and 3 with 2 and 4). FEN1 phosphorylation is described to initiate a proteasomal degradation program consisting of a sequential cascade of SUMOylation and subsequent ubiquitination [21]. However, experiments utilizing the protein synthesis inhibitor cycloheximide clearly revealed enhanced FEN1 protein stability in the presence of IE1 (see Fig 4). This led us to speculate that IE1 prevents proteasomal degradation of phosphorylated FEN1 species. Therefore, we compared the levels of pan-FEN1 and phosphorylated FEN1 in the presence of IE1 with those during inhibition of proteasomal degradation with MG132 (Fig 8G). 293T cells were transfected with the indicated plasmids and treated with or without MG132 for 6 hours before harvesting the cells. Interestingly, we could observe that, by performing three independent experiments, the addition of MG132 evokes a similar accumulation of phosphorylated FEN1 thereby indicating that IE1 could actually act by inhibiting the proteasomal degradation of phosphorylated FEN1 species (Fig 8H). These experiments clearly demonstrate that IE1 mediates an accumulation of phosphorylated FEN1 in transfection and infection experiments.

### FEN1 is required for IE1-mediated activation of γH2AX during HCMV infection

**Fig 9:**
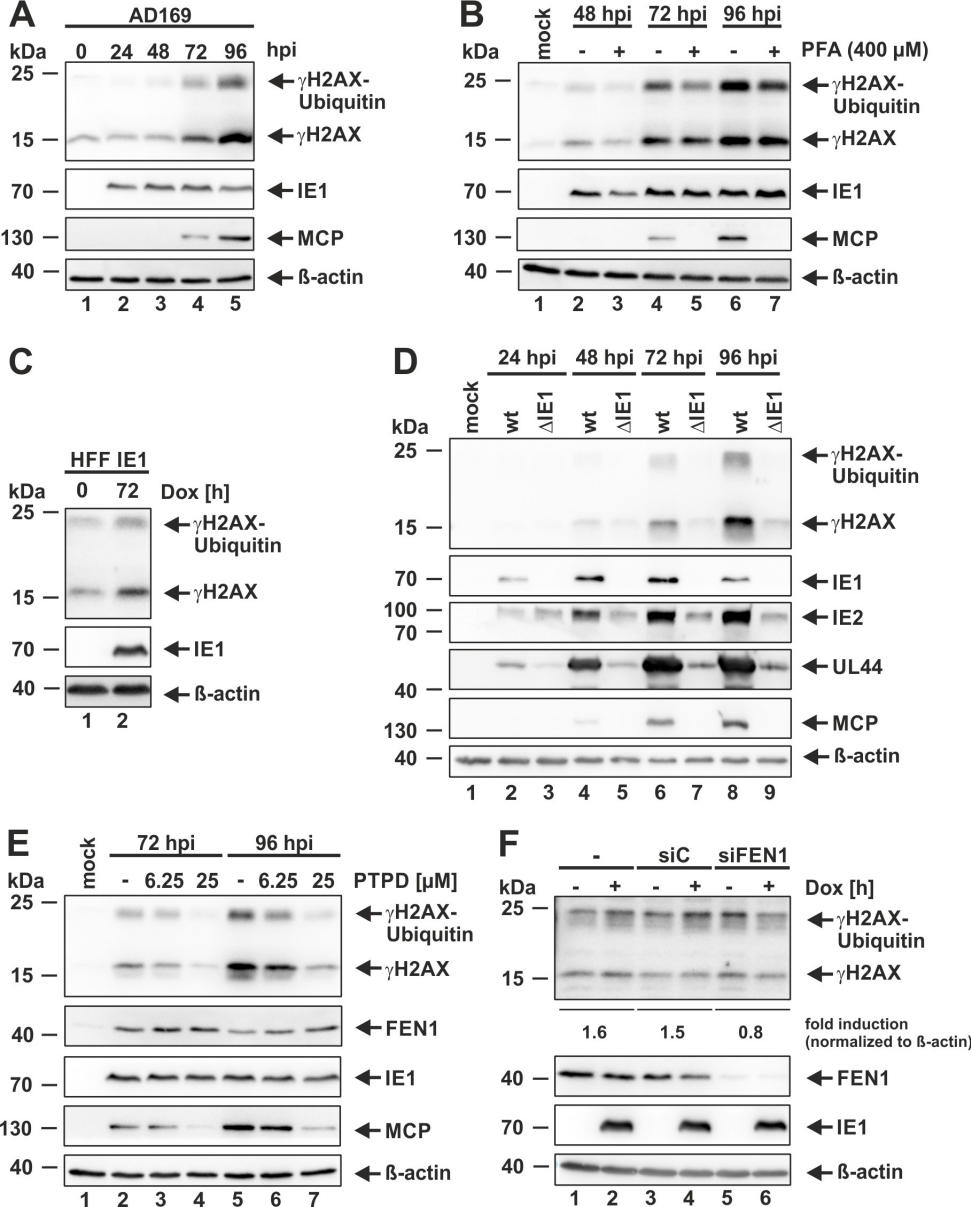
Requirement of FEN1’s enzymatic activity for the induction of γH2AX. (A, B, D, and E) HFF cells were either mock infected or infected with AD169 at an MOI of 3 (A, E), 1 (B), or with AD169 (wt) at an MOI of 7 and equivalent genome copy numbers of a recombinant strain lacking IE1 (ΔIE1) (D). Cells were treated at 2 hpi with 400 μM phosphonoformic acid (PFA) (B) or with the indicated concentration of the FEN1 inhibitor PTPD (E). At the indicated times post infection, cells were harvested and analyzed by Western blotting for the indicated proteins. (C and F) HFF IE1 cells with doxycycline-inducible expression of IE1 were treated with control siRNA (siC) or siRNA targeting FEN1 (siFEN1) when indicated (F) and either not induced or induced with 0.5 μg/ml doxycycline and analyzed by Western blotting for γH2AX, IE1, and β-actin.

As phosphorylation of FEN1 is described to stimulate its double strand break (DSB)-generating gap endonuclease activity [24], we propose that FEN1 might be involved in the induction of γH2AX, a sensitive marker for DSBs, during the late phase of HCMV infection [6–8] (Fig 9A). To address this issue, we pursued to analyze the expression of γH2AX in HCMV-infected cells in the presence of the FEN1 inhibitor PTPD. Beforehand, to exclude that γH2AX expression requires an active viral DNA replication, which is negatively affected by FEN1 inhibition (see Fig 6B), we analyzed γH2AX levels in the presence of the viral DNA synthesis inhibitor phosphonoformic acid (PFA). By comparing AD169-infected HFFs, which were PFA-treated or left untreated, it could be clearly demonstrated that the induction of γH2AX does not depend on viral DNA replication (Fig 9B, first panel, compare lanes 4 and 6 with 5 and 7), while expression of the late protein MCP was completely prevented (Fig 9B, third panel, compare lanes 4 and 6 with 5 and 7). Moreover, by utilizing doxycycline-inducible HFF IE1 cells, we confirmed the previous finding that IE1 alone is sufficient to activate γH2AX [8] (Fig 9C). To analyze whether IE1 is also necessary for γH2AX activation, we performed comparative analyses by infecting HFF cells with a high MOI of wild-type AD169 (wt) and equivalent genome copy numbers of a recombinant HCMV strain lacking IE1 (Fig 9D). While we could observe a robust induction of γH2AX in wild-type AD169 infected cells (Fig 9D, first panel, lanes 4, 6, and 8), cells infected with the HCMV strain lacking IE1 only displayed weak activation of γH2AX (Fig 9D, first panel, lanes 5, 7, and 9). Interestingly, similar results were obtained when applying the FEN1 inhibitor PTPD during AD169 infection (Fig 9E). A dramatic reduction of γH2AX could be detected in a dose-dependent manner (Fig 9E, first panel). This finding strongly indicates that FEN1 might be required for the IE1-driven activation of γH2AX (see Fig 9C). In order to address this issue, we repeated the experiment as performed for Fig 9C but additionally transfected either control siRNA (siC) or siRNA against FEN1 (siFEN1) (Fig 9F). By utilizing this approach, we could demonstrate that FEN1 is required for IE1-mediated γH2AX activation (Fig 9F, first panel, compare lanes 1 to 4 with lanes 5 and 6).

### FEN1 re-initiates stalled viral replication forks by inducing DSBs and γH2AX

**Fig 10:**
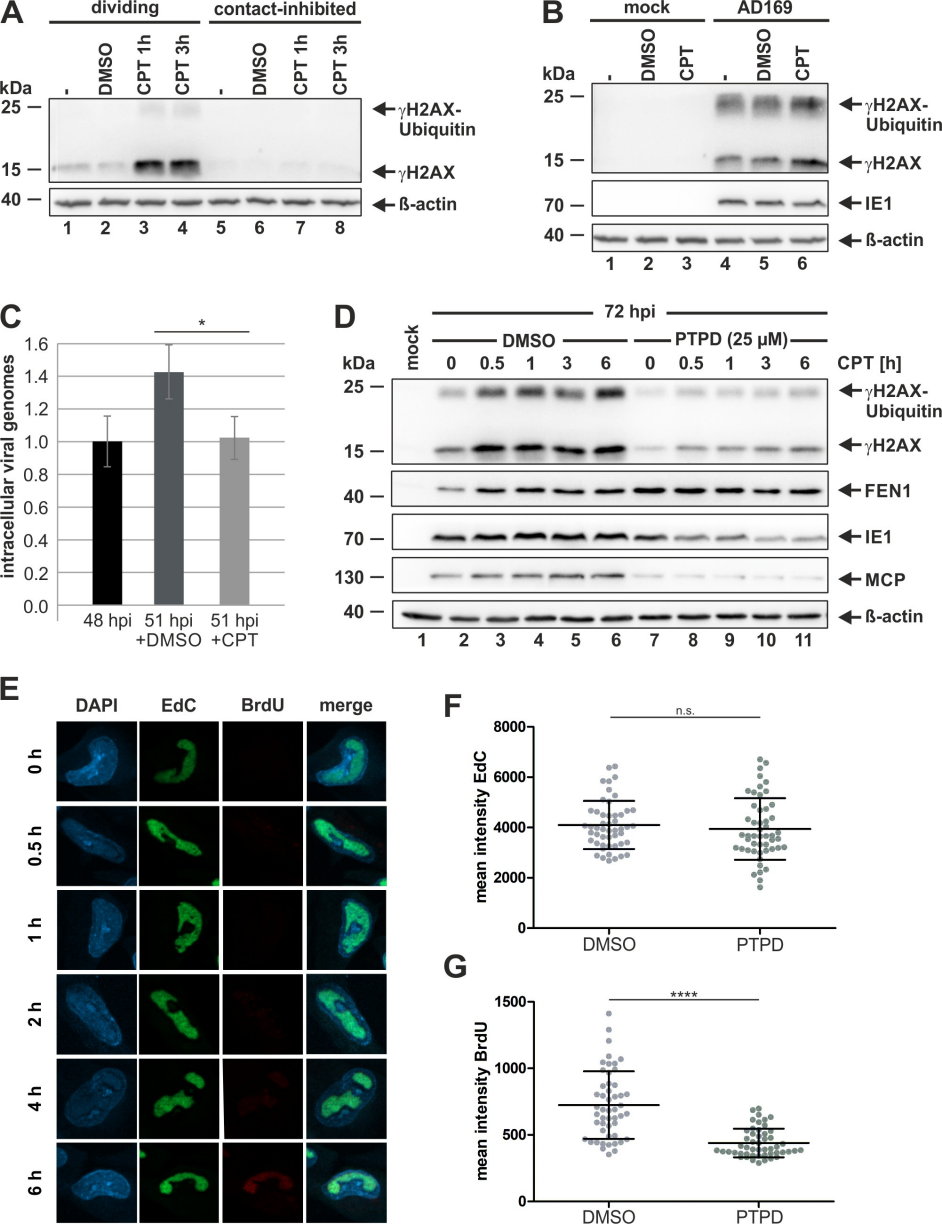
Requirement of FEN1’s enzymatic activity for the re-initiation of stalled viral replication fork. (A, B, and D) HFF cells were either mock infected (A, B, and D) or infected with AD169 at an MOI of 3 (B and D). When indicated, cells were treated with DMSO or PTPD (25 μM) at 2 hpi (D). DMSO or 1 μM camptothecin (CPT) were applied for 1 h unless otherwise indicated. Subsequently, cells were harvested and analyzed by Western blotting for the indicated proteins. (C) HFFs were infected with AD169 at an MOI of 0.1, and total DNA was extracted at 48 hpi (first bar) or at 51 hpi after a 1 h treatment with DMSO (second bar) or 1 μM CPT (third bar) using the DNeasy blood and tissue kit (Qiagen). Viral genomes were quantified by TaqMan real-time PCR specific for IE1, and genome copy numbers were calculated. HFFs infected for 48 h were set to 1. Values are derived from biological triplicates and represent mean values ± SD. (E) Visualization of viral DNA labeled with two synthetic nucleotides: EdC (second panel), which was incorporated before the induction of replication fork stalling with 1 μM CPT, and BrdU (third panel), which was incorporated after release from the CPT block. Time points reflect times after release. (F and G) Mean fluorescence intensities of incorporated EdC (F) or BrdU (G) quantified in 50 cells treated with DMSO (left) or 25 μM PTPD (right) 6 h after CPT release. For panels C, F, and G, the p-values were calculated using two-tailed Student’s t-tests. n.s., not significant; ****, p ≤ 0.0001; *, p ≤ 0.05.

As FEN1 is beneficial for viral DNA replication (see Figs 5 and 6), we suggested that the expression of γH2AX during HCMV expression might be indicative for DSBs, which are generated by FEN1 to re-initiate stalled viral replication forks. In order to answer this issue, we made use of the replication fork-stalling agent camptothecin (CPT) that has been shown to induce DSBs and γH2AX exclusively in S-phase, at the sites of DNA replication [40–44]. First, to exclude that we detect CPT-mediated induction of DSBs and γH2AX at cellular and not at viral replication forks, we checked whether CPT could induce γH2AX in contact-inhibited HFFs, since these conditions are used for HCMV infection experiments. Therefore, we treated HFFs that were either dividing or contact-inhibited with 1 μM CPT for the indicated times (Fig 10A). Remarkably, we could not detect any signal for γH2AX in contact-inhibited HFFs (Fig 10A, lanes 5 to 8), while CPT treatment in dividing cells induced a robust γH2AX signal indicative for DSBs at cellular replication forks (Fig 10A, lanes 1 to 4). Next, contact-inhibited HFFs were either mock-infected or infected with AD169 and, at 72 hpi, treated with 1 μM CPT (Fig 10B). Again, we could not detect any signal in contact-inhibited uninfected cells (Fig 10A, lanes 1 to 3), while AD169-infected cells displayed an induction of γH2AX, which is further enhanced following CPT treatment (Fig 10B, upper panel, lanes 3 to 6). This experiment unequivocally proves that γH2AX, induced by CPT during HCMV infection, reflects DSBs at the viral replication fork. In order to confirm that CPT is able to block viral DNA replication by inducing replication fork stalling, we quantified intracellular viral genomes by quantitative real-time PCR at 48 hpi before and 3 hours later after a short incubation with either the solvent control DMSO or CPT (Fig 10C). As depicted in Fig 10C, the addition of DMSO alone did not perturb viral DNA replication as indicated by a 1.4-fold increase in nascent viral genomes at 51 hpi (Fig 10C, compare first and second bar). In contrast, there was no increase in nascent viral genomes in the presence of CPT detectable (Fig 10C, compare first and third bar). Subsequently, we wanted to investigate whether FEN1 is required for the induction of DSBs and γH2AX at the viral replication fork upon treatment with CPT. For this, we infected contact-inhibited HFF, treated them with PTPD or the solvent control DMSO and, at 72 hpi, applied CPT for different periods of time to provoke replication fork stalling. Time course analyses showed reduced γH2AX induction in PTPD treated cells thereby suggesting that FEN1 activity is required to generate DSBs at the viral replication fork upon stalling (Fig 10D, compare lanes 7 to 11 with 2 to 6). Consequently, we continued to analyze the role of FEN1 for the re-initiation of stalled viral replication forks. For this, we established a protocol based on the sequential incorporation of synthetic nucleotides into newly synthesized DNA. This approach allows for visualization of replication fork progression on the cellular level via immunofluorescence. Therefore, HFFs were infected with AD169 and, at 48 hpi, treated with EdC to allow for incorporation into DNA, with the last one hour in the presence of the replication fork-stalling agent CPT. After a wash-out step, BrdU was added to the culture medium for different periods of times to visualize the re-initiation of replication forks that were stalled by CPT. Afterwards, BrdU and EdC were detected via immunostaining and click chemistry, respectively. As expected, both EdC and BrdU were observed in viral replication compartments (Fig 10E, second and third panel). However, while EdC is visible at all time points (Fig 10E, second panel), the BrdU signal emerges at 4 hours post addition thus reflecting the onset of replication fork re-initiation (Fig 10E, third panel). Accordingly, γH2AX levels in HCMV-infected and CPT treated cells were decreased 3 hours following release indicating that replication forks have undergone DSB repair (S2 Fig). Having successfully established a technique that allows for the detection of replication fork re-initiation, we finally pursued to compare BrdU incorporation between DMSO- and PTPD treated cells at 6 hours after CPT release by repeating the before mentioned procedure. Subsequently, mean fluorescence intensities of EdC and BrdU were measured in DMSO- and PTPD treated cells. Remarkably, there was no significant difference in EdC incorporation thus excluding variations in DNA synthesis before the induction of replication fork stalling with CPT (Fig 10F). In contrast, BrdU incorporation was significantly reduced in cells treated with PTPD in comparison to cells treated with the solvent control DMSO (Fig 10G). This experiment clearly demonstrates that FEN1 activity is involved in the re-initiation of stalled viral replication forks.

## Discussion

Meanwhile it is well documented that DNA viruses are able to engage components of the host DNA damage and repair machinery to ensure the success of their replicative programs [2]. So far, knowledge on the utilization of the structure-specific endonuclease FEN1 during viral infections is limited. Only recently FEN1 was recognized as a key enzyme for hepatitis B virus cccDNA formation and as a marker for hepatitis B virus-associated hepatocellular carcinoma [45–49]. This emphasizes that blocking enzymatic activities of DNA repair proteins such as FEN1 may represent novel therapeutic opportunities for the treatment of viral diseases [50]. In this study we provide evidence that human cytomegalovirus, a ubiquitous herpesvirus and important pathogen in immunosuppressed patients, manipulates FEN1 in a unique way to foster the re-initiation of stalled viral replication forks.

The relationship between human cytomegalovirus (HCMV) and the cellular DNA damage response (DDR) has been discussed in a controversial manner during the last years. It could be shown that HCMV infection triggers the ATM branch of DDR, which responds to DNA double strand breaks (DSBs) by activating homologous recombination (HR)-mediated repair pathways [11]. However, while one report identified this activation to be necessary for efficient viral DNA replication [8], other groups postulated that DDR is not required for viral DNA replication and that the responses downstream of ATM are subverted by the mislocalization of repair proteins [6,7]. Interestingly, there is increasing evidence that the HCMV major immediate-early protein IE1 acts as a main driver for DDR activation and, moreover, is even able to stimulate HR-mediated repair [8,11,12]. Thus, we propose that cellular DNA repair processes can be actively induced by viral proteins in order to promote replication of the viral genome.

Therefore, it was an affirmatory finding for us to identify three proteins among 12 involved in DNA repair processes when searching for novel cellular IE1 interactors in a yeast two-hybrid based approach (Fig 1A). While the protein Adenine DNA glycosylase is important for the initiation of base excision repair, Homologous-pairing protein 2 homolog and Flap endonuclease 1 (FEN1) are involved in HR-mediated repair processes [13,51,52]. As interaction with the latter was readily confirmed by co-immunoprecipitation analysis in transfected as well as infected cells (Fig 2A, 2B and 2C), we continued to investigate the role of FEN1 for the replication of HCMV.

We found FEN1 to be upregulated starting with the early phase of HCMV infection (Fig 3A and 3B), where viral DNA replication takes place, thereby suggesting a possible involvement of FEN1 in viral DNA replication. As FEN1 physically interacts with IE1, we consequently analyzed whether IE1 is responsible for the enhanced protein expression of FEN1. By utilizing a doxycycline-inducible system, we could reliably demonstrate that an isolated expression of IE1 is sufficient for the upregulation of FEN1 (Fig 3C). Experiments with a recombinant virus strain lacking IE1 revealed that IE1 is even necessary for the upregulation of FEN1 at the initial phase of infection (Fig 3D). The promoter region of FEN1, which represents a typical S-phase protein, harbors a consensus site for and is bound by the transcription factor E2F1 that is described as critical determinant for the cellular G_1_/S-phase transition [53]. Interestingly, IE1 itself has been shown to induce the expression of E2F regulated genes by inactivating p107 and p130, which are both repressors of E2F proteins [54–56]. Infection experiments utilizing an siRNA directed against E2F1 could show that E2F1 contributes to the upregulation of FEN1 suggesting a regulation at the transcriptional level (Fig 3E). Moreover, experiments with the protein synthesis inhibitor cycloheximide revealed that IE1 binding increases FEN1 protein stability thereby providing an explanation for IE1-mediated FEN1 upregulation during HCMV infection (Fig 4).

In order to elucidate the role of FEN1 for lytic HCMV replication, we generated primary human fibroblasts harboring a knockdown of FEN1 and analyzed the growth dynamics of HCMV in these cells. We detected a significant yet mild delay of viral growth (Fig 5B), which may be explained by the incomplete knockdown of FEN1 after stable expression of an shRNA (Fig 5A). The delay of HCMV growth on cells lacking FEN1 was further demonstrated to depend on the multiplicity of infection (MOI) showing the highest requirement for FEN1 at low MOI conditions (Fig 5C). This is in accordance with the MOI-dependent growth properties of recombinant IE1-deleted HCMV strains thereby suggesting that the IE1-FEN1 interplay might be one determinant for an efficient HCMV growth at low MOIs [57,58]. Interestingly, this is not the first observation that FEN1 is hijacked in order to promote viral replication: FEN1 is not only implicated to be a critical enzyme for hepatitis B virus cccDNA formation, but it is also involved in the synthesis and maturation of herpes simplex virus type 1 lagging-strand intermediates [47,59]. As FEN1 is upregulated with early kinetics (Fig 3A and 3B), we speculated that FEN1 might be beneficial for viral DNA replication. Indeed, we were able to identify an active role of FEN1 during viral DNA replication since depletion of FEN1 protein resulted in a reduced number of nascent viral genomes (Fig 5E and 5F). To exclude that FEN1 might solely act as a kind of recruiting or stabilizing factor for other proteins—without requiring its own enzymatic activity—we utilized the FEN1 inhibitor PTPD followed by the quantification of intracellular viral genomes. Strikingly, we could detect a 2-fold reduction of viral genomes when inhibiting the enzymatic activity of FEN1 (Fig 6), which is entirely consistent with results obtained in depletion experiment (Fig 5E and 5F).

FEN1 is a multifunctional nuclease that participates in distinct DNA metabolic pathways by exerting different nuclease activities. In order to prevent damage of the cellular genome, FEN1 needs to be tightly regulated in a spatiotemporal manner via interplaying mechanisms: protein turnover, formation of complexes with different protein partners, post-translational modifications and subcellular compartmentalization [16,45,60]. To obtain further insight how FEN1 acts to promote viral DNA replication, we analyzed the subcellular localization of FEN1 during HCMV infection by utilizing HFFs stably expressing mCherryFEN1 (S1 Fig). Surprisingly, we found FEN1, which displays a pan-nuclear staining pattern in uninfected cells, to be excluded from the nucleolus starting with the immediate-early phase of infection (Fig 7A and 7B). By utilizing cells with doxycycline-inducible expression of IE1, we could pin down this translocation to be directly mediated by IE1 (Fig 7C). In contrast, the FEN1 binding-deficient mutant IE1 5x mut (see Fig 2D and 2E) was not able to alter the localization of FEN1 thereby demonstrating the requirement of a direct protein-protein interaction (Fig 7D). In 2008, Guo and colleagues described this translocation event to be mediated by UV-irradiation that damages the DNA and potentially stalls replication forks [27]. The authors speculated that FEN1, which is physiologically enriched in nucleoli for ribosomal DNA replication, is translocated out of nucleoli upon DNA damage to participate in the re-initiation of stalled DNA replication forks [27]. Moreover, they could show that UV-induced phosphorylation at Ser187 signals the protein to translocate itself from the nucleolus to the nuclear plasma [27]. In accordance with this study, we could consistently detect enhanced accumulation of FEN1 phosphorylated at Ser187 in the presence of IE1 wt but not the FEN1 binding-deficient mutant IE1 5x mut (Fig 8A, 8B and 8D). As we found FEN1 likewise phosphorylated during HCMV infection (Fig 8E), we asked whether IE1 might be necessary for the accumulation of phosphorylated FEN1 species. Comparative analyses between an HCMV wildtype strain and a recombinant mutant strain lacking IE1 confirmed our hypothesis, as we could not detect FEN1 phosphorylation during infection with the IE1-deleted virus (Fig 8F). However, we are not able to rule out that early or late proteins, which were severely decreased during mutant virus infection, might additionally influence FEN1 phosphorylation. It has previously been shown that, under physiological conditions at the end of the S-phase, phosphorylation of Ser187 initiates a posttranslational modification program consisting of SUMO3 modification and subsequent ubiquitination resulting in proteasomal degradation of FEN1 [21]. In contrast, a more recent study by Xu *et al*. reported that UV irradiation and exposure to the fork-stalling agents hydroxyurea, camptothecin and mitomycin C induces sequential FEN1 phosphorylation at Ser187 and SUMOylation by SUMO1, which was not associated with ubiquitination and proteasomal degradation [21,25]. To analyze the fate of FEN1 in the presence of IE1, we performed experiments with the protein synthesis inhibitor cycloheximide in order to examine the stability of FEN1 (Fig 4). FEN1 alone was observed to be quite unstable (Fig 4A), as already shown by Guo *et al*. [21]. Surprisingly, FEN1 underwent a tremendous increase of stability in presence of IE1 wt (Fig 4B), but not in presence of the binding-deficient IE1 5x mut (Fig 4C). This result strongly argues against the proteasomal degradation scenario initiated by phosphorylation and SUMO3 modification. Consequently, we speculated that IE1 might even prevent proteasomal degradation. To answer this issue, we compared the levels of pan-FEN1 as well as phosphorylated FEN1 in presence of either IE1 or MG132, which serves as inhibitor of the proteasome. Strikingly, we observed with both treatments an equal accumulation (2.5-fold) of phosphorylated FEN1 species in comparison to pan-FEN1 (Fig 8G and 8H). The overall results strongly indicate that IE1 acts downstream of FEN1 phosphorylation by interfering with the proteasomal degradation program, which could be explained by different scenarios: abrogation or alteration of SUMO modification, abrogation of ubiquitination, or direct inhibition of the proteasome. Since a direct IE1-FEN1 interaction is required for its increased stability (compare Fig 4B and 4C), we would argue against an IE1-mediated inhibition of the proteasome. In line with this, IE1 was not yet described to prevent degradation of cellular proteins. In contrast, it was reported that IE1 can induce proteasomal degradation of the cellular restriction factor Sp100, the gap junctional protein Cx43 as well as the transcriptional regulator Hes1 [61–63]. Thus, IE1 could act either via abrogation of SUMOylation or ubiquitination. Both modifications are described to be subjected to highly reversible and dynamic pathways consisting of SUMO/ubiquitin ligases on the one hand and SUMO/ubiquitin proteases on the other hand [64]. Structural analyses of IE1, however, provide no evidence for the presence of a potential active site with hydrolase activity, which would be needed for acting as SUMO or ubiquitin protease [65]. Alternatively, IE1 might recruit proteases in order to activate them. However, the performed yeast two-hybrid based approach (see Fig 1A) as well as a further study did not support this idea [66]. Therefore, we would propose an alternative scenario. IE1 is described to oligomerize thereby providing an enlarged surface [65]. In this context, it has already been shown that IE1 interferes with the *de novo* SUMOylation of the cellular restriction factor PML via tight binding by utilizing its enlarged surface [67]. Thus, it is conceivable that IE1 acts in a similar way in order to prevent SUMOylation or ubiquitination of FEN1, which in turn would inhibit proteasomal degradation. In addition to the enhanced stability of FEN1, an accumulation of phosphorylated FEN1 was detected in the presence of IE1 (Fig 8A and 8B) and during HCMV infection (Fig 8E). Considering that FEN1 species phosphorylated at Ser187 are the preferred targets for the proteasomal degradation program, we would suppose that the observed accumulation of phosphorylated FEN1 is evoked by the IE1-mediated inhibition of this program [21]. In accordance, we could observe a comparable accumulation of phosphorylated FEN1 when directly inhibiting the proteasome (Fig 8G and 8H).

Taken together, our study provides insight on how FEN1 promotes HCMV genome replication. Phosphorylation of FEN1 has already been shown to stimulate gap endonuclease activity that is important for the active induction of DNA double strand breaks (DSBs), which in turn serves as initial step for the re-initiation of stalled replication forks by homologous recombination (HR)-mediated repair [16,24,25]. Interestingly, IE1 itself has been implicated in the induction of HR-mediated repair [12]. Therefore, we propose that IE1 induces the gap endonuclease activity of FEN1 in order to re-initiate stalled replication forks by inducing DSBs. We performed further experiments demonstrating that IE1 and FEN1 are both required for the efficient induction of DSBs during HCMV infection, as indicated by γH2AX (Fig 9D and 9E). Moreover, we could show that IE1 is even able to stimulate FEN1 in uninfected cells in order to generate DSBs (Fig 9F). Those results motivated us to analyze the role of FEN1 for the re-initiation of stalled viral replication forks. For this, we made use of the stalling agent camptothecin (CPT), which was analyzed concerning its impact on viral replication forks: CPT efficiently stimulates viral replication fork stalling, as indicated by a complete block of viral DNA replication (Fig 10C), which was followed by the induction of DSBs at the viral replication fork (Fig 10B). The CPT-mediated induction of DSBs was shown to depend on FEN1 (Fig 10D). By utilizing an immunofluorescence-based approach that allows for the sequential incorporation of synthetic nucleotides, we could finally prove that FEN1 is required to re-initiate stalled viral replication forks (Fig 10E, 10F and 10G).

In line with this, the genome of HCMV has been reported to be G/C-rich (57.2%) with some regions of very high G/C content. Therefore, it can be considered as difficult-to-replicate since GC-rich regions tend to form secondary structures like G-quadruplexes resulting in replication fork stalling [68,69]. Remarkably, FEN1 has previously been described to facilitate DNA replication at difficult-to-replicate regions, including rDNA and telomeres [27,70,71]. Thus, we propose the G/C-rich genome of HCMV as novel substrate for FEN1 that is activated by IE1 in order to re-initiate stalled replication forks. Stunningly, it was reported that a smaller IE1 protein species (IE1x4) recruits the enzyme TOPOIIβ, which is also known to induce DSBs [72,73], to the GC-rich TR element in latently infected cells and that HCMV latent DNA replication requires the activity of TOPOIIβ [74]. Thus, HCMV may have evolved several independent mechanisms to ensure its replicative success during both lytic and latent infection.

## Material and methods

### Oligonucleotides and plasmids

The oligonucleotide primers used for this study were purchased from Biomers GmbH (Ulm, Germany) and are listed in Table 1.

**Table 1:** Oligonucleotides.

5’pQEHisStrep_IE1_EcoRI	CATAGAATTCATGGAGTCCTCTGCCAAGAG
3’-IE1-GEX-Sal	TCACGTCGACTTACTGGTCAGCCTTGCTTCTAG
5’IE1_aa14_EcoRI	CATAGAATTCCCTGACGAGGGCCCTTCCTCC
3’IE1_aa382_SalI	CATAGTCGACTTACTCTTCCTCATCTGACTCCTC
3’ IE1aa1-359_SalI	CATAGTCGACTCAATCGGCCCCCAGAATGTACTG
5’IE1mutY206A	GGAGAAAGATGATGGCTATGTGCTACAGG
3’IE1mutY206A	CCTGTAGCACATAGCCATCATCTTTCTCC
5’IE1mutK223A	GAACTCAGCCTTCCCTGCGACCACCAATGGCTGCAG
3’IE1mutK223A	CTGCAGCCATTGGTGGTCGCAGGGAAGGCTGAGTTC
5’IE1mutN237A	CATGGCGGCACTGCAGGCCTTGCCTCAGTGCTCC
3’IE1mutN237A	GGAGCACTGAGGCAAGGCCTGCAGTGCCGCCATG
5’IE1mutN285A	GTGGAAACAATGTGTGCTGAGTACAAGGTCAC
3’IE1mutN285A	GTGACCTTGTACTCAGCACACATTGTTTCCAC
5’IE1mutM296A	CTAGTGACGCTTGTATGGCGACCATGTACGGGGGC
3’IE1mutM296A	GCCCCCGTACATGGTCGCCATACAAGCGTCACTAG
5’IE1mutR310A	CTTAAGTGAGTTCTGTGCGGTGCTGTGCTGCTATG
3’IE1mutR310A	CATAGCAGCACAGCACCGCACAGAACTCACTTAAG
5’pQEHisStrep1/IE1	CATAGGATCCATGGAGTCCTCTGCCAAGAG
3’IE1aa382_EcoRI	CATAGAATTCTTACTCTTCCTCATCTGACTCCT
Top_siFEN1	GATCCGGGAGAATGACATCAAGAGTTCAAGAGACTCTTGATGTCATTCTCCCTTTTTTACGCGTG
Bottom_siFEN1	AATTCACGCGTAAAAAAGGGAGAATGACATCAAGAGTCTCTTGAACTCTTGATGTCATTCTCCCG
mCherry_PacI_fw	CATATTAATTAAATGGTGAGCAAGGGCGAGG
mCherry_PacI_rev	CATATTAATTAACTTGTACAGCTCGTCCATGC
5’NsiI_FEN1	CATAATGCATATGGGAATTCAAGGC
3’MfeI_FEN1	CATACAATTGTCATTTTCCCCTTTTAAAC
5’attB1/IE1	GGGGACAAGTTTGTACAAAAAAGCAGGCTATGGAGTCCTCTGCCAAGAG
3’attB2/IE1	GGGGACCACTTTGTACAAGAAAGCTGGGTCTTACTGGTCAGCCTTGCTTC
c-CRS-mut	GCGTGTACGGTGGGAGGCCTATATAAGCAGAGCCTAGGTAGGGAGAAGTCAGATCGCCTGGAGACGCC
nc-CRS-mut	GGCGTCTCCAGGCGATCTGACTTCTCCCTACCTAGGCTCTGCTTATATAGGCCTCCCACCGTACACGC
5’ FEN1 S187A mut	CCTCACCTTCGGCGCCCCTGTGCTAATGC
3’ FEN1 S187A mut	GCATTAGCACAGGGGCGCCGAAGGTGAGG
5’ CMV	AAGCGGCCTCTGATAACCAAG
3’ CMV	GAGCAGACTCTCAGAGGATCGG
CMV MIE FAM/TAMRA	CATGCAGATCTCCTCAATGCGGCG
5’ Alb	GTGAACAGGCGACCATGCT
3’ Alb	GCATGGAAGGTGAATGTTTCAG
Alb FAM/TAMRA	TCAGCTCTGGAAGTCGATGAAACATACGTTC

The constructs expressing IE1 FL, IE1 14–382, and IE1 1–359, all in fusion with the GAL4 DNA-binding domain, were generated by amplification of IE1 (pHM494) with primers 5’pQEHisStrep_IE1_EcoRI + 3’-IE1-GEX-Sal, 5’IE1_aa14_EcoRI + 3’IE1_aa382_SalI, 5’pQEHisStrep_IE1_EcoRI + 3’ IE1aa1-359_SalI, respectively, followed by insertion of the yielded fragments into pGBT9 (Clontech, Mountain View, CA, USA). Plasmids expressing IE1 (pHM494), FLAG-IE1 (pHM3289), FLAG-IE1 1–382 (pHM3362), and Myc-IE1 1–382 (pHM3589) were generated as described elsewhere [30,65,75]. For transient expression of FLAG-FEN1 176–380 and Myc-FEN1 176–380, the FEN1 fragment, which was identified by yeast two-hybrid screening, was amplified by utilizing primers 5’Not_Fen1_aa176 + 3’Xba_Fen1 and inserted into pHM971 (pcDNA3.1-FLAG) and pHM1580 (pcDNA3.1-Myc), respectively [30,31]. An expression plasmid for FLAG-FEN1 was constructed by amplifying FEN1 from pShuttle-FEN1hWT, a gift from Sheila Stewart (Addgene plasmid # 35027; http://n2t.net/addgene:35027; RRID:Addgene_35027), with primers 5’NotI_FEN1 + 3’Xba_Fen1 and by inserting it into pHM971 (pcDNA3.1-FLAG) [30,71]. Derivatives of IE1 with point mutations in conserved or exposed residues (Y206A mut, Loop mut, Helix 8 mut, 5x mut) were constructed either by Invitrogen GeneArt Site-Directed Mutagenesis System (Thermo Fisher Scientific, Waltham, MA, USA) according to the manufacturer’s instructions (Y206A mut, 5x mut (K223A, N237A, N285A, M296A, R310A)) or by subcloning a synthetic IE1 gene, in which the respective nucleotides were exchanged, into pHM494 (Loop mut (aa 50–55 LDGPLF to GSGSGS), Helix 8 mut (E334A, S337A, V338S, K340E)). For the latter approach, the synthetic cDNA of IE1 Loop mut and -Helix 8 mut was synthesized by GeneArt gene synthesis service (Thermo Fisher Scientific, Waltham, MA, USA) and fragments carrying either the Loop mutation or the Helix 8 mutation were subcloned into pHM494 via HindIII/AflII or AflII/EcoRI, respectively. For generating constructs expressing Myc-tagged IE1 1–382 versions harboring the distinct mutations, the above-mentioned constructs were utilized for amplification with primers 5’pQEHisStrep1/IE1 + 3’IE1aa382_EcoRI, followed by insertion into pHM1580 (pcDNA3.1-Myc) [31]. The pSIREN-RetroQ FEN1 plasmid was constructed by annealing oligosTop_siFEN1 + Bottom_siFEN1 and inserting the annealed oligos coding for shRNA against FEN1 into vector pSIREN-RetroQ (Clontech, Takara Bio Inc., Kusatsu, Japan) via BamHI/EcoRI. The pSIREN-RetroQ-based construct expressing siC was generated as described elsewhere [76]. To generate mCherry- and mCherryFEN1-expressing plasmids, mCherry was amplified from the plasmid pRSET-B-mCherry (a kind gift from Roger Y. Tsien) with primers mCherry_PacI_fw + mCherry_PacI_rev and inserted into the already described modified pLKO-based lentiviral vector [67]. For generation of a mCherryFEN1 expressing plasmid, the yielded construct was cleaved by NsiI/EcoRI, followed by insertion of FEN1, which was amplified from the plasmid expressing FLAG-FEN1 with primers 5’NsiI_FEN1 + 3’MfeI_FEN1. To construct pInducer20-based plasmids (pInducer20 was a gift from Stephen Elledge (Addgene plasmid # 44012; http://n2t.net/addgene:44012; RRID:Addgene_44012)) with inducible expression of IE1 wt or IE1 5x mut, the respective coding sequences were amplified from either pHM494 or the above described IE1 5x mut plasmid, respectively, with primers 5’attB1/IE1 + 3’attB2/IE1. The resulting PCR products were transferred into the intermediate vector pDONR221 and subsequently introduced into a modified version of the lentiviral expression vector pInducer20 (pInducer20 CRS mut) using the Invitrogen Gateway recombination technology (Thermo Fisher Scientific, Waltham, MA, USA). pInducer20 CRS mut was constructed by utilizing Invitrogen GeneArt Site-Directed Mutagenesis System (Thermo Fisher Scientific, Waltham, MA, USA) according to the manufacturer’s instructions with primers c-CRS-mut and nc-CRS-mut. Constructs for transient expression of mutants S187A and S187D were generated with the plasmid expressing FLAG-FEN1 as template by using the GeneArt site-directed mutagenesis kit as instructed by the manufacturer (Thermo Fisher Scientific, Waltham, MA, USA).

### Yeast two-hybrid analyses

*Saccharomyces* cerevisiae Y153 (His^-^, Leu^-^, Trp^-^) was used in a two-hybrid system. Both the plasmid pGBT9 (Clontech, MountainView, CA, USA) encoding the GAL4-DB (Trp^+^) fusion protein and the plasmid pGAD424 (Clontech, MountainView, CA, USA) encoding the GAL4-A (Leu^+^) fusion protein were introduced into Y153 cells using a modified lithiumacetate (LiAc) method [77]. For this, cells were grown overnight in YAPD medium, pelleted and treated with LP-mix (40% w/v PEG4000, 0.15 M LiAc, 10 mM Tris/HCl pH 7.5, 1 mM EDTA pH 8.0) and DMSO. Single-stranded carrier-DNA as well as both plasmids were added to the yeast cells. This step was followed by incubation at room temperature and subsequent incubation at 42°C. Thereafter, the cells were plated on WL^-^ minimal selection agar. For rapid in situ assays of lacZ expression from yeast colonies, an XGal filter assay was used [78]. Nitrocellulose filters were laid onto the plate and allowed to wet completely, then lifted off the plate and placed in liquid nitrogen to permeabilize the cells. The filters were removed and placed cell side up in a petri dish containing Whatman Paper soaked with Z buffer containing ß-Mercaptoethanol and XGal. The filters were incubated at 30°C and constantly analyzed for the development of a positive blue color.

### Yeast two-hybrid screening

Yeast two-hybrid screening was performed with GAL4 fusion proteins as described previously [28]. Yeast strain Y153 was transformed by the LiAc method by using the globular core region of IE1 (IE1_CORE_), which comprises amino acids 14 to 382, as bait in fusion with the DNA-binding domain of the transcription factor GAL4 in the pGBT9 vector backbone. The GAL4-BD (Trp^+^) fusion encoding IE1_CORE_ was stably maintained in yeast strain Y153 (His^-^, Leu^-^, Trp^-^) by selection in liquid culture minimal medium lacking Trp. The yeast two-hybrid screen was performed by transformation of yeast strain Y153 containing IE1_CORE_ in fusion with the Gal4-BD with a cDNA library derived from B lymphocytes in the pACT (Leu^+^) vector backbone [29]. Directly after transformation, yeast cells were plated on HWL^-^ minimal selection agar. These plates enable selection of transformants harboring both plasmids (leucine- and tryptophan prototrophy) as wells as of transformants bearing an interaction partner of IE1_CORE_ by selection for histidine prototrophy. Leaky expression of the HIS3 gene product was avoided by supplementation with 10 mM 3-aminotriazole. After 5 to 10 days, positive clones (indicated by a colony size ≥ 3 mm) were utilized for qualitative XGal filter lift assays [78]. The DNA of putative interactors was isolated by utilizing the Zymoprep Yeast Plasmid Miniprep Kit (Zymo Research, Irvine, CA, USA) and sequenced using a primer specific for the pACT plasmid. Subsequently, the DNA was transformed into the E.coli strain KC8 and plated on M9 minimal agar supplemented with tryptophan to isolate the pACT plasmid carrying the LEU2 gene.

### Cells and viruses

HEK293T cells were cultivated in Dulbecco’s minimal essential medium (DMEM) (Thermo Fisher Scientific, Waltham, MA, USA) containing 10% fetal calf serum (FCS). Primary human foreskin fibroblasts (HFFs) were prepared from human foreskin tissue and were maintained in Eagle’s minimal essential medium (MEM) (Gibco, Thermo Fisher Scientific, Waltham, MA, USA) supplemented with 7% fetal calf serum. HFFs with inducible expression of IE1 were cultured in MEM (Gibco, Thermo Fisher Scientific, Waltham, MA, USA) supplemented with 10% tetracycline-free fetal bovine serum (Clontech, Palo Alto, CA, USA), 5 μg/ml puromycin, and 500 μg/ml Geneticin. HFFs with a small interfering RNA-mediated knockdown of FEN1 (siFEN1) and control HFFs (vector, siC) were cultured in MEM (Gibco, Thermo Fisher Scientific, Waltham, MA, USA) supplemented with 7% fetal calf serum and 1 μg/ml puromycin. Infection experiments were performed with HCMV laboratory strain AD169, the recombinant virus AD169ΔIE1, and clinical isolate TB40/E at specified multiplicities of infection (MOIs) [37,79]. Viral stocks were titrated via IE1p72 fluorescence [80]. For this purpose, HFFs were infected with various dilutions of virus stocks. After 24 h of incubation, cells were fixed and stained with monoclonal antibody (MAb) p63-27, directed against IE1p72. Subsequently, the number of IE1-positive cells was determined and used to calculate viral titers, expressed as IE protein-forming units (IEU).

### Transfection and doxycycline induction

HEK293T cells were transfected with plasmid DNA either by applying the standard calcium phosphate coprecipitation method or by utilizing the TurboFect transfection reagent according to instructions of the manufacturer (Thermo Fisher Scientific, Waltham, MA, USA). For this, 7x10^5^ HEK293T cells were seeded into six-well dishes. One day after seeding, cells were transfected with 2 to 4 μg of plasmid DNA, depending on the experiment. When utilizing the calcium phosphate coprecipitation method, the cells were washed two times with phosphate-buffered saline without calcium and magnesium and provided with fresh medium 16 h after transfection. At 48 h after transfection, cells were harvested for further analyses. To test protein stability, the protein synthesis inhibitor cycloheximide (CHX) was applied 18 h after transfection at a concentration of 10 μg/ml. Subsequently, cells were harvested at the indicated time points after CHX addition. Transfection with siRNAs was performed by seeding 1x10^5^ HFFs in 12-well dishes and, one day later, applying 50 nM siRNA directed against FEN1 (sc-37795; Santa Cruz Biotechnology, Santa Cruz, CA, USA) or E2F1 (L-003259-00-0005; Horizon Discovery, Cambridge, UK) as well as 2 μl Lipofectamine 2000 reagent (Invitrogen, Karlsruhe, Germany) in the presence of OptiMEM (Gibco, Thermo Fisher Scientific, Waltham, MA, USA). After 4 to 6 h, the medium was replaced by normal growth medium. At 24 h after transfection, cells were infected with AD169 and harvested at 96 hpi for further analyses. For the induction of IE1 expression, HFF-IE1 cells were treated with 0.5 μg/ml doxycycline for indicated times and harvested for Western blot or immunofluorescence analyses.

### Antibodies

Monoclonal antibodies used for immunofluorescence and Western blot analyses were: α-IE1 63–27 [81], α-UL44 BS510 (kindly provided by B. Plachter, Mainz, Germany), α-MCP 28–4 [82], α-FLAG M2 (Sigma-Aldrich, Deisenhofen, Germany), α-Myc 9E10, α-β-actin AC-15 (Sigma-Aldrich, Deisenhofen, Germany), α-FEN1 B-4 (Santa Cruz Biotechnology, Santa Cruz, CA, USA), α-phospho-Histone H2A.X (Ser139) (20E3) (Cell signaling technology, Danvers, MA, USA), α-E2F1 (KH95) (Santa Cruz Biotechnology, Santa Cruz, CA, USA) and α-BrdU MoBu-1 (Thermo Fisher Scientific, Waltham, MA, USA). The polyclonal antibodies α-phospho FEN1 (Ser187) (Thermo Fisher Scientific, Waltham, MA, USA) and α-p-Histone H2A.X (Ser 139) sc-101696 (Santa Cruz Biotechnology, Santa Cruz, CA, USA), and were used for Western blot analyses. Secondary antibodies used for immunofluorescence and Western blot analyses were: Alexa Fluor 488-/555-/647-conjugated secondary antibodies for indirect immunofluorescence experiments were purchased from Molecular Probes (Karlsruhe, Germany), horseradish peroxidase-conjugated anti-mouse/-rabbit secondary antibodies for Western blot analyses were obtained from Dianova (Hamburg, Germany).

### Immunoblotting

Lysates from infected, transduced, transfected or induced cells were prepared in a sodium dodecyl sulfate-polyacrylamide gel electrophoresis (SDS-PAGE) loading buffer, separated on SDS-containing 8 to 15% polyacrylamide gels, and transferred to nitrocellulose membranes. Chemiluminescence was detected according to the manufacturer’s protocol (ECL Western blot detection kit; Amersham Pharmacia Biotech).

### Coimmunoprecipitation

Transfected HEK293T cells (1.4x10^6^) or infected HFFs expressing mCherry/mCherryFEN1 (1x10^6^) were lysed for 20 min at 4°C in 800 μL of CoIP buffer (50 mM Tris-HCl [pH 8.0], 150 mM NaCl, 5 mM EDTA, 0.5% NP-40, 1 mM PMSF, 2 μg/mL of aprotinin, 2 μg/mL of leupeptin, and 2 μg/mL of pepstatin). After centrifugation, aliquots of each sample were taken as input controls and the remaining supernatant was incubated with anti-FLAG antibody M2 coupled to protein-A-sepharose beads or anti-mCherry antibody 16D7 (Thermo Fisher Scientific, Waltham, MA, USA) coupled to protein-G-dynabeads for 2 h at 4°C. The beads were collected by centrifugation and washed five times in 1 mL CoIP buffer. Finally, the immunoprecipitated proteins were recovered by boiling in 4xSDS sample buffer and protein complexes were analyzed by immunoblotting.

### Indirect immunofluorescence

HFF cells grown on coverslips in six-well dishes (3x10^5^ cells/well) were washed twice with PBS at indicated times. Cells were fixed with a 4% paraformaldehyde solution for 10 min at room temperature (RT) and then washed for two times. Permeabilization of cells was achieved by incubation with 0.2% Triton X-100 in PBS on ice for 20 min. Cells were washed again with PBS over a time period of 5 min and incubated with the appropriate primary antibody diluted in PBS-1% FCS for 30 min at 37°C. Excessive antibodies were removed by washing four times with PBS, followed by incubation with the corresponding fluorescence-coupled secondary antibody diluted in PBS-1% FCS for 30 min at 37°C. The cells were mounted using the DAPI-containing Vectashield mounting medium (VECTOR LABORATORIES, Burlingame, CA, USA) and analyzed using either a Leica TCS SP5 confocal microscope with the 543-nm laser line or an Axio-Observer.Z1 fluorescence microscope (Carl Zeiss Microscopy GmbH) with 469/38 nm, 555/30 nm, 631/33 nm LED sources.

### Generation of lentiviruses and transduction of HFFs

The transduction of HFFs with lentiviruses derived from the pLVX-Tet-On Advanced vector and lentiviruses derived from pLVX-Tight-Puro-IE1 in order to yield HFFs with inducible expression of IE1 were generated as described elsewhere [37]. For generation of HFF cells stably expressing mCherry and mCherryFEN1 or HFFs cells stably expressing inducible IE1 versions, replication-deficient lentiviruses were generated using pLKO-based or pInducer20-based expression vectors. For this purpose, HEK293T cells seeded in 10-cm dishes (4.9x10^6^ cells) were cotransfected with pLKO vectors encoding mCherry and mCherryFEN1 or pInducer20 vector encoding IE1 versions together with packaging plasmids pLP1, pLP2, and pLP/VSV-G using the Lipofectamine 2000 reagent (Invitrogen, Karlsruhe, Germany). Viral supernatants were harvested 48 h after transfection, cleared by centrifugation, filtered, and stored at -80°C. HFFs were incubated for 24 h with lentiviral supernatants in the presence of 7.5 μg/ml polybrene (Sigma-Aldrich, St. Louis, MO, USA). Stably transduced HFF cell populations were selected by adding 500 μg/ml Geneticin to the cell culture medium.

### Generation of retroviruses and transduction of HFFs

Replication-deficient, murine leukemia virus-based retroviruses were prepared by cotransfection of HEK293T cells (4.9x10^6^) with a pSIREN-RetroQ FEN1 plasmid together with packaging plasmids pHIT60 (kindly provided by K. Überla, Erlangen, Germany) and pVSV-G using the Lipofectamine 2000 reagent (Invitrogen, Karlsruhe, Germany). Viral supernatants were harvested 48 h after transfection, clarified by centrifugation, filtered, and stored in aliquots at -80°C. Low-passage-number primary HFF cells were incubated for 24 h with retrovirus supernatants in the presence of 7.5 μg/ml of Polybrene (Sigma-Aldrich, St. Louis, MO, USA). Then, puromycin (5 μg/ml) was added to the cell culture medium in order to select a stably transduced cell population. Additionally, control cells were prepared in parallel using control retroviruses expressing either no siRNA (vector) or a nonfunctional siRNA (siC).

### Multistep growth curve analysis

HFF cells were seeded into six-well dishes at a density of 3x10^5^ cells/well and infected the following day with wild-type AD169 at a multiplicity of infection (MOI) of 0.01. Triplicate samples of the infected cell supernatants were harvested at 2, 4, 6, 8, and 10 days after inoculation and subjected to lysis by proteinase K (Sigma-Aldrich, St. Louis, MO, USA) for 1 h at 56°C, followed by an inactivation step for 5 min at 95°C. Afterwards, quantitative real-time PCR (TaqMan-PCR) of a sequence region within exon 4 of the IE1 gene locus was conducted as described below to analyze the amount of viral genome copies in the supernatants.

### Quantitative TaqMan real-time PCR

A total of 3x10^5^ infected HFF cells were used in triplicates for TaqMan real-time PCR. For quantification of intracellular viral genomes, total DNA was extracted using the DNeasy blood and tissue kit according to the manufacturer’s instructions (Qiagen, Hilden, Germany). For assessing viral genome copy numbers released from infected HFFs, the cell culture supernatants were collected, centrifuged at 1,500 × g, and treated with proteinase K (Sigma-Aldrich, St. Louis, MO, USA) for 1 h at 56°C, followed by an inactivation step for 5 min at 95°C. Next, 5 μl from each sample (virus-containing supernatant treated with proteinase K or extracted intracellular DNA) were utilized for quantitative real-time PCR (TaqMan PCR), which was conducted as described previously [37]. To quantify viral DNA, a sequence within the major immediate early gene region was amplified using the primers 5′CMV and 3′CMV as well as the labeled probe CMV MIE FAM/TAMRA. For analysis of intracellular genomes, quantification of cellular albumin genes was performed in parallel using the primers 5′ Alb and 3′ Alb along with the labeled probe Alb FAM/TAMRA.

### Cell viability assay

The determination of cell viability was performed using the CellTiter-Glo Luminescent Cell Viability Assay (Promega, Madison, WI, USA) according to the manufacturer’s instructions. Therefore, 1 x10^4^ HFF cells were seeded in 96-well plates and treated with the indicated substances for 96 h. Subsequently, luminescent signal was recorded in an appropriate plate reader.

### Replication fork re-initiation assay

HFF cells grown on coverslips in 12-well dishes (1.2x10^5^ cells/well) were infected at a MOI of 1 and, at 2 hpi, treated with the solvent control DMSO or PTPD (25 μM). 48 hpi, 1 μM 5-ethynyl-2’-deoxycytidine (EdC) was added to the culture medium for approximately 16 h to label newly synthesized DNA, with the last 1 h in the presence of 1 μM camptothecin (CPT). After washing-out EdC and CPT, 10 μM BrdU, provided in warm culture medium containing either DMSO or PTPD, was added to the cells. Newly synthesized DNA is subsequently labeled with BrdU not EdC, which is ensured by extensive washing steps and a 10-fold molar excess of BrdU. At the indicated times post BrdU addition, cells were washed twice with PBS and fixed with ice-cold methanol/glacial acetic acid (3:1) for 10 min on ice and then washed for three times with PBS. DNA denaturation was achieved by incubation with 4N HCl for 10 min at room temperature. Cells were washed again, once with A.d. and additional four times with PBS to neutralize HCl. The conjugation of Alexa Fluor 488 azide to EdC-labeled DNA was performed by click chemistry using 10 mM sodium ascorbate and 1 mM CuSO_4_ for 2 h at room temperature. Subsequent BrdU detection was obtained via immunostaining. After a 1 h blocking step with 5% BSA in 0.1% Triton/PBS, we incubated cells for 2 h at 37°C with the anti-BrdU clone MoBu-1 (diluted in 0.5% BSA in 0.1% Triton/PBS) that exclusively detects BrdU- bot not EdC-labeled DNA. Excessive antibodies were removed by washing four times with PBS, followed by incubation with secondary antibody Alexa Fluor 555 diluted in 0.5% BSA in 0.1% Triton/PBS for 30 min at 37°C. The cells were mounted using the DAPI-containing Vectashield mounting medium (VECTOR LABORATORIES, Burlingame, CA, USA) and analyzed using an Axio-Observer.Z1 fluorescence microscope (Carl Zeiss Microscopy GmbH) with 469/38 nm and 555/30 nm LED sources.

## Supporting information

S1 Fig Generation of HFFs stably expressing mCherry and mCherryFEN1. (A and B) Detection of mCherryFEN1 after lentiviral transduction by indirect immunofluorescence analyses detecting the red fluorescent protein mCherry (A) or by Western blotting utilizing an antibody directed against FEN1 (B).
(TIF)

S2 Fig Decrease of γH2AX in HCMV-infected cells following release from CPT block. HFF cells were infected with AD169 at an MOI of 1, treated, at 48 hpi, with DMSO or 1 μM CPT, and released for the indicated times from CPT block. Cell were harvested and analyzed by Western blotting for the indicated proteins.
(TIF)
