# Supplementary figures and images for: Functional regulation of the structure-specific endonuclease FEN1 by the human cytomegalovirus protein IE1 suggests a role for the re-initiation of stalled viral replication forks
Source: PLoS Pathog. 2021 Mar 26;17(3):e1009460. doi: 10.1371/journal.ppat.1009460 (PMC8026080; doi:10.1371/journal.ppat.1009460)

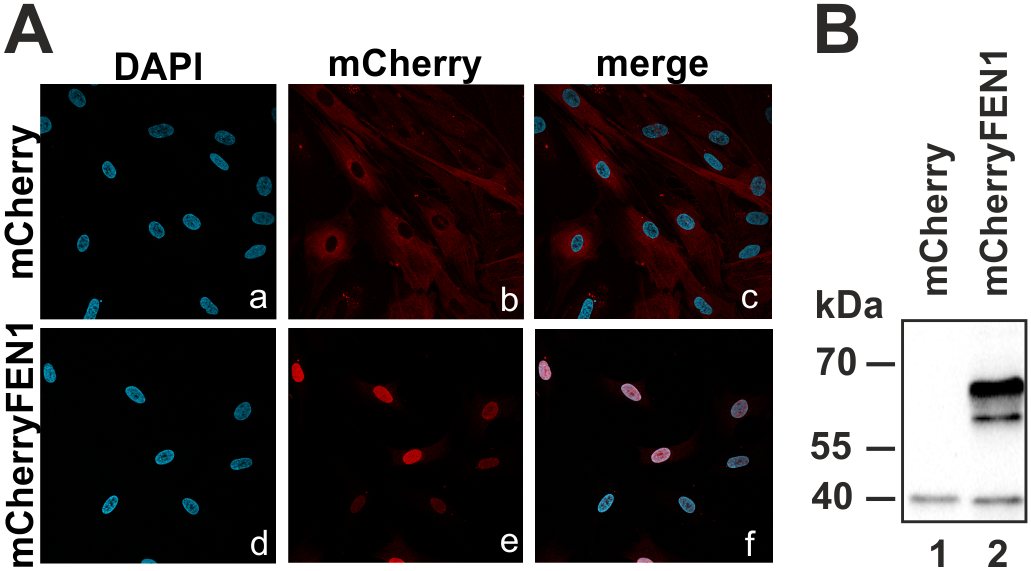

Supplement: S1 Fig — (A and B) Detection of mCherryFEN1 after lentiviral transduction by indirect immunofluorescence analyses detecting the red fluorescent protein mCherry (A) or by Western blotting utilizing an antibody directed against FEN1 (B). (TIF) [file ppat.1009460.s001.tif]

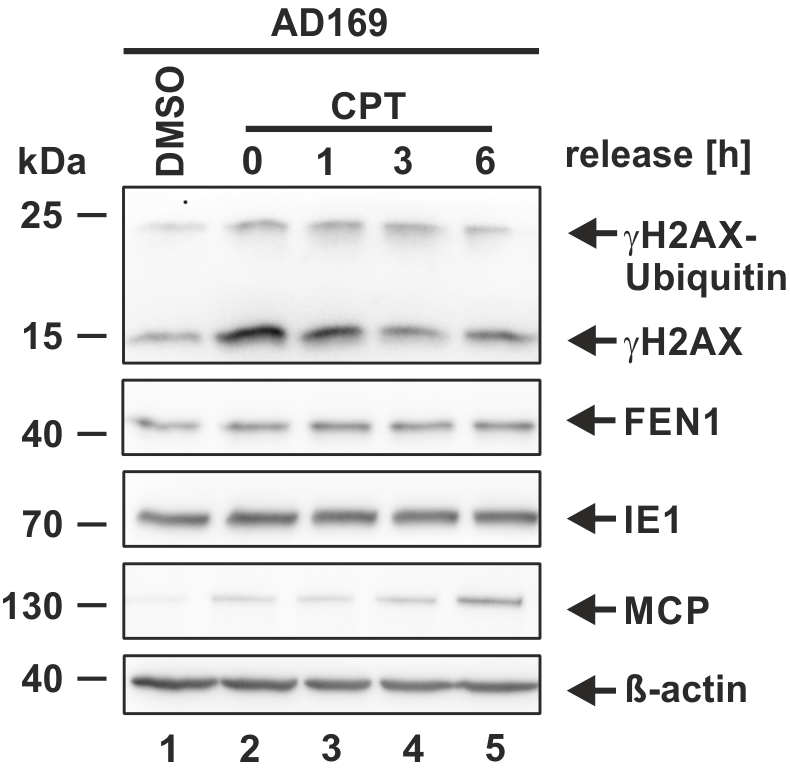

Supplement: S2 Fig — HFF cells were infected with AD169 at an MOI of 1, treated, at 48 hpi, with DMSO or 1 μM CPT, and released for the indicated times from CPT block. Cell were harvested and analyzed by Western blotting for the indicated proteins. (TIF) [file ppat.1009460.s002.tif]
